# A constrained mixture-micturition-growth (CMMG) model of the urinary bladder: Application to partial bladder outlet obstruction (BOO)

**DOI:** 10.1016/j.jmbbm.2022.105337

**Published:** 2022-06-30

**Authors:** Fangzhou Cheng, Paul N. Watton, Giulia Pederzani, Masahiro Kurobe, Ei-ichiro Takaoka, Chris Chapple, Lori Birder, Naoki Yoshimura, Anne M. Robertson

**Affiliations:** a Department of Mechanical Engineering and Materials Science, University of Pittsburgh, Pittsburgh, United States; b Department of Urology, University of Pittsburgh, Pittsburgh, United States; c Sheffield Teaching Hospitals NHS Foundation Trust, Sheffield, United Kingdom; d Department of Medicine, University of Pittsburgh, United States; e Department of Computer Science & Insigneo Institute for in silico Medicine, University of Sheffield, Sheffield, United Kingdom

**Keywords:** Bladder, Model, Growth, Remodeling, Biomechanics, Mechanobiology, Bladder outlet obstruction

## Abstract

We present a *constrained mixture-micturition-growth* (CMMG) model for the bladder. It simulates bladder mechanics, voiding function (micturition) and tissue adaptations in response to altered biomechanical conditions. The CMMG model is calibrated with both *in vivo* and *in vitro* data from healthy male rat urinary bladders (cystometry, bioimaging of wall structure, mechanical testing) and applied to simulate the growth and remodeling (G&R) response to partial bladder outlet obstruction (BOO). The bladder wall is represented as a multi-layered, anisotropic, nonlinear constrained mixture. A short time scale micturition component of the CMMG model accounts for the active and passive mechanics of voiding. Over a second, longer time scale, G&R algorithms for the evolution of both cellular and extracellular constituents act to maintain/restore bladder (homeostatic) functionality. The CMMG model is applied to a spherical membrane model of the BOO bladder utilizing temporal data from an experimental male rodent model to parameterize and then verify the model. Consistent with the experimental studies of BOO, the model predicts: an initial loss of voiding capacity followed by hypertrophy of SMC to restore voiding function; bladder enlargement; collagen remodeling to maintain its role as a protective sheath; and increased voiding duration with lower average flow rate.

This CMMG model enables a mechanistic approach for investigating the bladder’s structure–function relationship and its adaption in pathological conditions. While the approach is illustrated with a conceptual spherical bladder model, it provides the basis for application of the CMMG model to anatomical geometries. Such a mechanistic approach has promise as an *in silico* tool for the rational development of new surgical and pharmacological treatments for bladder diseases such as BOO.

## 1. Introduction

The function of the bladder is to serve as a low pressure reservoir for storing urine and then efficiently expelling this urine at a convenient time. Bladder outlet obstruction (BOO) is a urodynamic diagnosis that signifies the existence of increased urethral resistance, sufficient to alter the voiding process. Over time, BOO can lead to changes in both the bladder’s storage capacity and functionality giving rise to lower urinary tract symptoms (LUTs) that dramatically lower quality of life, both physically and psychologically (Nitti, 2005).

Clinical diagnosis of BOO is based on the presence of specific changes to the bladder’s pressure flow relationship, as defined in the International Continence Society (ICS) Standardization report, and can be measured using urodynamic studies (I.C. Society, 2021). The mechanical causes include an enlarged prostate, such as induced by benign prostatic hyperplasia (BPH) (Patel and Parsons, 2008, 2014; Reddy and Shaik, 2019) and urethral narrowing from scarring or strictures (Groutz et al., 2000; Tritschler et al., 2013; Alwaal et al., 2014). BPH induced BOO is increasingly prevalent for men over the age of 50, with 50%–75% of men over age 50 and 80% of men over age 70 experiencing LUTS (voiding hesitancy, prolonged micturition, incomplete bladder emptying, increased urination frequency, urgency, and incontinence) as a result of this condition (Egan, 2016). The economic burden is significant, e.g., 4 billion dollars annually in the US in 2006 (Taub and Wei, 2006). Moreover, with a worldwide prevalence of over 210 million, it is an increasingly important global health issue (Trail et al., 2021) and as the population ages and men enjoy increased life expectancy, the impact of BPH will continue to rise (Launer et al., 2021).

The structure of the bladder wall is often considered with respect to three layers: the mucosa; the muscularis propria, herein referred to as the detrusor smooth muscle layer (DL); and the adventitia (AD). The mucosa is the innermost layer and is composed of a multi-cell layer epithelium (termed urothelium), a basement membrane and a lamina propria (LP) which contains a densely packed, interwoven network of collagen fibers. The DL is a composite of smooth muscle cell (SMC) bundles intermixed with collagen and elastin fibers and the outer surface of the bladder is formed of loose connective tissue commonly termed the adventitia. During filling, the unfolding of the tissue layers allow the bladder to expand under low pressure (Hornsby et al., 2017; Cheng et al., 2018). To void, smooth muscle cells are triggered to generate active stress and initiate flow by overcoming the urethral resistance.

The bladder, like other soft tissues can alter its constituents and geometry through a growth and remodeling (G&R) process. In bladders with BOO, the urethral resistance increases, requiring the SMC in the wall to generate larger pressures to void, inducing a G&R response that leads to changes in bladder size, tissue composition and functionality (Damaser et al., 1999) in both humans and animal models (Fusco et al., 2018; Oelke et al., 2002). However, whilst this G&R response can restore voiding, it can lead to deficits in mechanical function and associated LUTs. Experimental and computational models of the G&R response can provide insight into the evolving pathophysiology and potentially offer improvements to diagnosis, management and treatment of BOO.

Over the past 20 years, constrained mixture (CM) models of soft-tissue G&R have been developed to study tissue adaptations during disease and in response to injury or clinical intervention (Humphrey, 2021). However, thus far, no models have been applied to simulate the evolution of bladder disease.

In this work, experimental data from a male rat model of BOO and corresponding sham data are used to drive an integrative *in vivo*, *in vitro* and *in silico* modeling approach for studying BOO. In particular, we develop a theoretical model of the bladder that combines a CM model of the tissue with a micturition model and uses rate-based G&R algorithms for adaption of the extra-cellular matrix and SMCs to BOO. We refer to this as a *constrained-mixture-micturition-growth* (CMMG) model and apply it to model micturition in the healthy bladder and its G&R in response to outlet obstruction, for illustration, using an idealized spherical model of the bladder. BOO is simulated by an increase in outlet resistance and competing hypotheses for the bladder’s adaptive response to restore its voiding functionality are investigated. Where possible, model parameters are informed from *in vivo* pressure-flow experiments and *in vitro* planar biaxial testing coupled with multiphoton microscopy to quantify the recruitment of collagen fibers and temporal changes to the bladder following BOO surgery. This modeling approach provides an investigative tool to explore the adaptive response of the bladder to altered (patho-)physiology and facilitates interpretation of the coupling between urodynamic curves and the underlying microstructure.

The structure of the paper is as follows: Section 2 details the experimental model, protocols for tissue characterization and provides a theoretical presentation of the CMMG model; Section 3 provides results of the *in vivo* and *in vitro* analysis of sham and BOO bladders. Section 4 describes the results of the *in silico* simulation of sham and BOO bladders using the CMMG model. Lastly, Section 5 compares model predictions with experimental observations, critiques the model and provides an outlook for future research.

## 2. Materials and methods

**Fig. 1.**
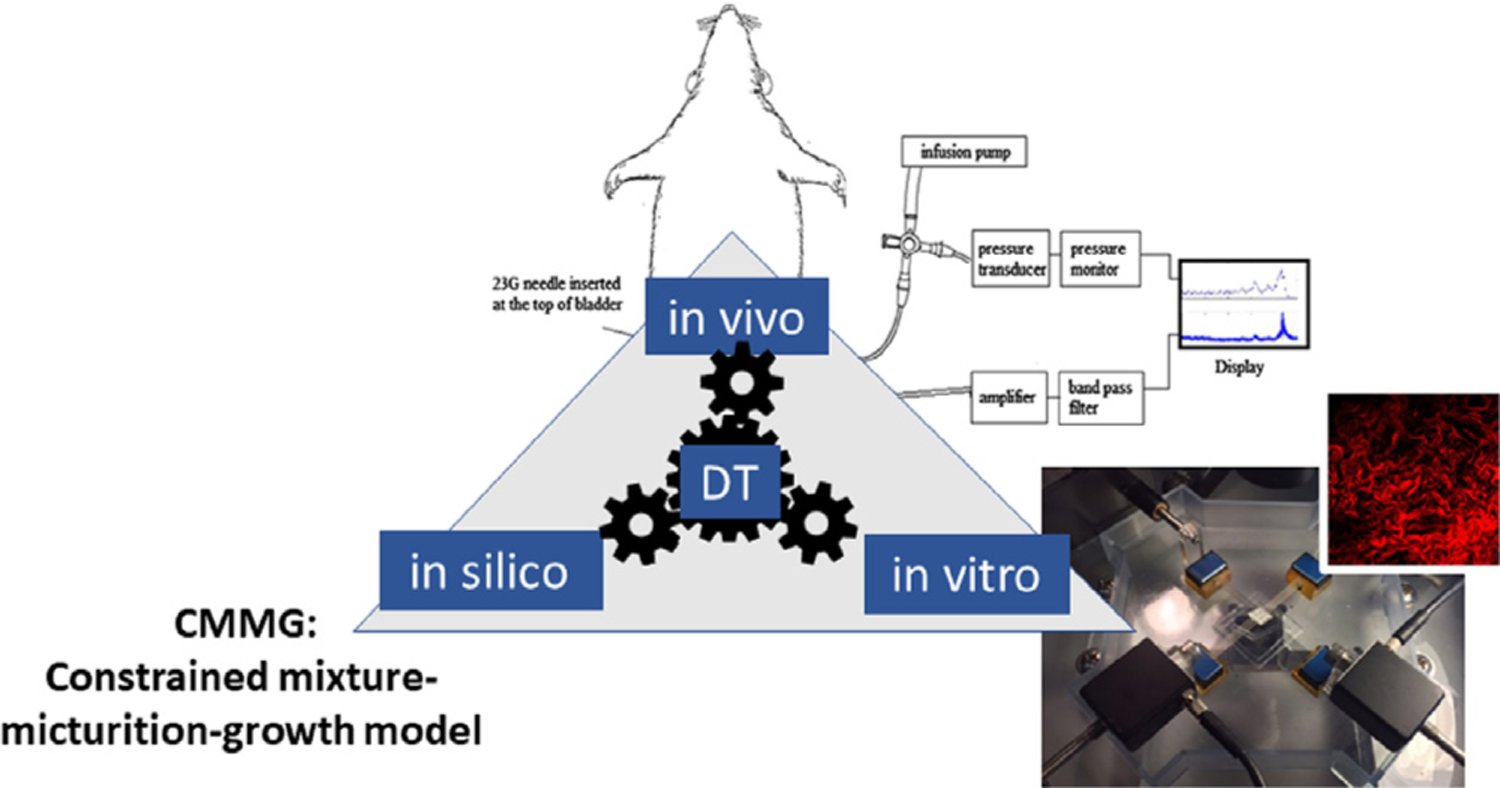
An integrative *in vivo*, *in vitro* and *in silico* modeling approach.

An overview of the integrative methods taken in this work are shown schematically in Fig. 1. Sections 2.1 and 2.2 overview the *in vivo* and *in vitro* experimental work, respectively. Methods for the CMMG model are distributed as follows: constitutive modeling 2.3; micturition modeling 2.4; homeostasis and G&R 2.5; numerical implementation for the conceptual bladder model using idealized spherical geometry and isotropic response 2.6.

### 2.1. In vivo testing

#### 2.1.1. Creation of sham and BOO rat models

Male Sprague Dawley rats were used for producing the experimental BOO model (Zvara et al., 2002). Briefly, under isoflurane anesthesia, the bladder and the proximal urethra were exposed via a lower abdominal midline incision. A 4-0 silk ligature was placed around the urethra and tied at the urethrovesical junction level proximal to the urethral fenestration with a metal rod (outside catheter diameter of 1.27 mm) placed alongside the urethra, and then the rod was removed. The abdominal wound was closed. This ligation was maintained in place throughout the duration of the experiments. Sham rats underwent similar procedures without urethral ligation.

#### 2.1.2. In vivo: Pressure-flow studies

Twenty four young (3-month old) male rats weighing 370 ± 8.9*g*, were used in the cystometry (pressure-flow) studies using previously established methodologies (Zvara et al., 2002). Measurements were performed 4 weeks after inducing BOO (n=12) as well as for rats with a sham surgery (n=12). Institutional Animal Care and Use Committee (IACUC) guidelines were observed. Briefly, under isoflurane anaesthesia, a PE-50 polyethylene catheter (Clay-Adams, Parsippany, NJ) was inserted through the bladder dome and a purse-string suture was placed tightly around the catheter. The implanted catheter was exteriorized through the abdominal wall, and the wound was closed with 4-0 silk sutures. The rats were placed in restraining cages (W 80 mm ×L 300 mm ×H 150 mm, Yamanaka Chemical Ind., Ltd, Osaka, Japan) and allowed to recover from isoflurane anaesthesia for 1–2 h before starting cystometry. After recovery, a three-way stopcock was used to connect the intravesical catheter to a pressure transducer (Transbridge 4M, World Precision Instruments, Sarasota, FL, USA) for recording intravesical bladder pressure and to a syringe pump (Harvard Apparatus,Holliston,MA) for infusing saline at a fixed flowrate. Because variability in bladder capacity among BOO rats is typical of this model even when the same obstruction technique was used, the saline injection speed was adjusted to maintain similar micturition intervals among animals. In particular, saline was initially infused at 0.1 ml/min; subsequently, the infusion rate was adjusted to 0.04–0.3 ml/min to obtain an intercontraction interval of approximately 10–15 min (Nishiguchi et al., 2007). Intravesical pressure changes were measured using data acquisition software (AD Instruments, Castle Hill, NSW, Australia) at a sampling rate of 100 Hz using a PowerLab. Saline infusion was continued until stable voiding cycles were established.

**Fig. 2.**
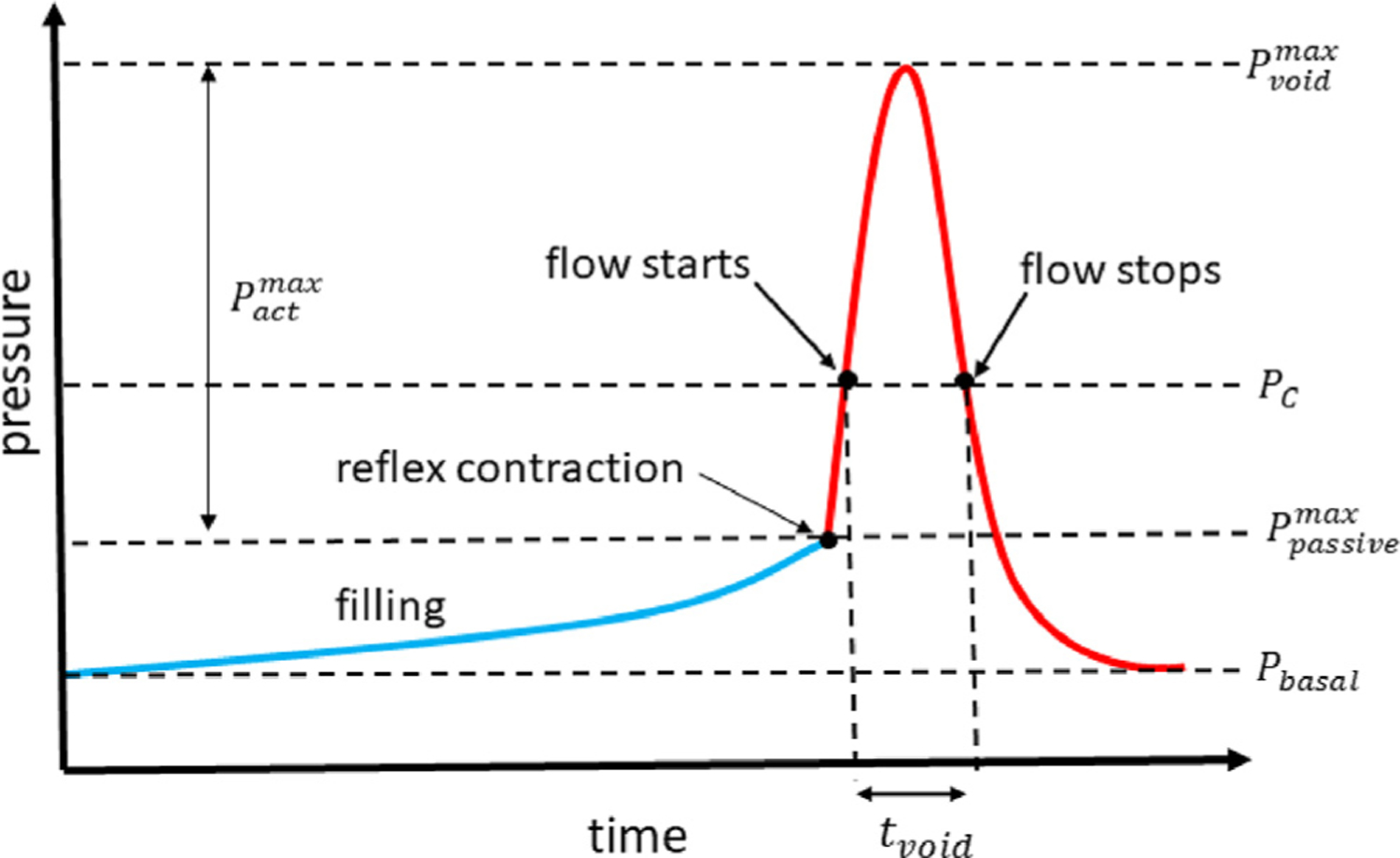
Schematic representation of the pressure flow curve during the cystometry studies. The bladder fills under low pressure. Following, the reflex contraction, the SMC develops actives stress and the bladder pressure increases. Initially, the bladder undergoes an isovolumetric contraction until the pressure exceeds the cut-off pressure *P_c_* at which point flow is initiated; flow stops when the bladder pressure reduces below *P_c_*. Immediately, following obstruction, the cut-off pressure increases and the bladder is unable to generate sufficient pressure to actively void and becomes leaky. Subsequent SMC hypertrophy increases the active pressure the bladder can generate and acts to restore voiding functionality.

The recorded data was used to construct the pressure curves from which the following parameters were evaluated: (i) maximum voiding pressure $P_{\text{void}}^{max}$; (ii) maximum passive filling pressure $P_{\text{passive}}^{max}$; (iii) maximum active voiding pressure $P_{act}^{max}$; see Table 1 for definitions and Fig. 2 for a illustrative pressure-curve for a healthy bladder. The voided urine was collected using a plastic cup placed underneath the restraining cage and measured to determine the voided volume (*V*_*void*_). The voiding duration, *t*_*void*_, was determined through direct observation of urine expelled from the urethral orifice. The post-void residual volume (*V*_*res*_) was measured by draining the post-void bladder using the bladder catheter with gravity (Takaoka et al., 2018). Bladder capacity, or filled volume (*V*_*F*_), was calculated as the sum of voided and residual volumes.

### 2.2. In vitro testing

Four sham and four partially obstructed (BOO) bladders were harvested at 4 weeks post surgery. To inhibit SMC contraction (Cheng et al., 2018), the dissected intact bladders were then immersed in Hank’s buffer salt solution (HBSS) containing, (in *mM*) NaCl 138, KCl 5, KH_2_ PO_4_ 0.3, NaHCO_3_ 4, MgCl_2_ 1, HEPES 10, glucose 5.6, with pH 7.4, 310 *mOsm/l*) without calcium and with added EDTA (0.5 *mM*). The voltage calcium channel blocker nifedipine (5μM; Sigma) and the SERCA pump inhibitor, thapsigargin (1μM; Tocris Biosciences), which prevents the reloading of intracellular calcium stores, were also added.

#### 2.2.1. In vitro: Bladder geometry

Harvested (unloaded) bladders resembled prolate spheroids and their outer dimensions, diameter (*D*_0_) and height (*Y*_0_), were measured. The bladders were subsequently cut open longitudinally and trimmed into square pieces with widths of 6 ± 1 mm such that the sides of the samples were aligned with the *in situ* longitudinal and circumferential directions. The unloaded thickness *H*_0_ of these samples were measured at 5 locations using a digital caliper (0–150 mm range, Marathon Watch Company Ltd) and averaged. The internal volume of the unloaded bladders (*V*_0_) were computed assuming a prolate spheroid geometry.

#### 2.2.2. In vitro: Biaxial testing and multi-photon microscopy

Mechanical testing was performed on square samples from four sham and four obstructed rat bladders using a custom biaxial system specifically designed for testing bladder tissue concurrent with imaging under a multiphoton microscope (MPM) (Cheng et al., 2018). This approach enables imaging of the reorganization and recruitment of collagen fibers during loading within fresh, intact specimens without staining or fixation. Fiducial strain markers (Basalt microspheres, 425–500 μm, Whitehouse Scientific) were attached to the abluminal side of each sample for strain calculations. The square samples were then mounted lumen side up in the biaxial device using biorakes (World Precision Instruments, Inc.) under the multiphoton system (Cheng et al., 2018). During testing, displacement was controlled by four actuators (Aerotech, Inc., linear actuator ANT-25LA) and force measurements were taken using load cells on two of the actuators (Transducer Techniques, nonrepeatability 0.05% of rate output, capacity 5 lbs). A CCD camera and a 45° offset mirror enabled imaging displacement of fiducial markers from beneath the mounted tissue (Cheng et al., 2018).

Following Wognum et al. (2009) and Cheng et al. (2018), load taring of 0.02N was applied to the sample after which it was pre-conditioned, then unloaded, then loaded to the tare load, and then mechanically tested. Five consecutive equibiaxial loading cycles to a prescribed sample stretch of 1.8 were performed for preconditioning, followed by five consecutive main loading cycles. Following Cheng et al. (2018), at stepwise increases in stretch, samples were scanned using a Z step size of 2 μm. Briefly, a multiphoton microscope (Olympus FV1000 MPE) with a Coherent Chameleon Ti-sapphire pulsed laser with 1.12 NA 25 ×MPE water immersion objective were used for all samples. Signals from second harmonic generation (SHG) were collected using a 400 nm emission filter with a ± 50 nm spectral bin for an excitation wavelength of 800 nm. As the penetration depth for MPM imaging for bladder tissue is less than the bladder thickness, the sample was imaged from both lumen and abluminal sides to assess collagen recruitment as a function of applied stretch. To avoid tissue damage at large strains, loading was stopped when collagen fibers were visibly straightened under MPM. Following earlier work (Wognum et al., 2009; Cheng et al., 2018), the Green–Lagrange strains and Cauchy stress were obtained from this data.

#### 2.2.3. In vitro: Collagen fiber recruitment distribution

**Fig. 3.**
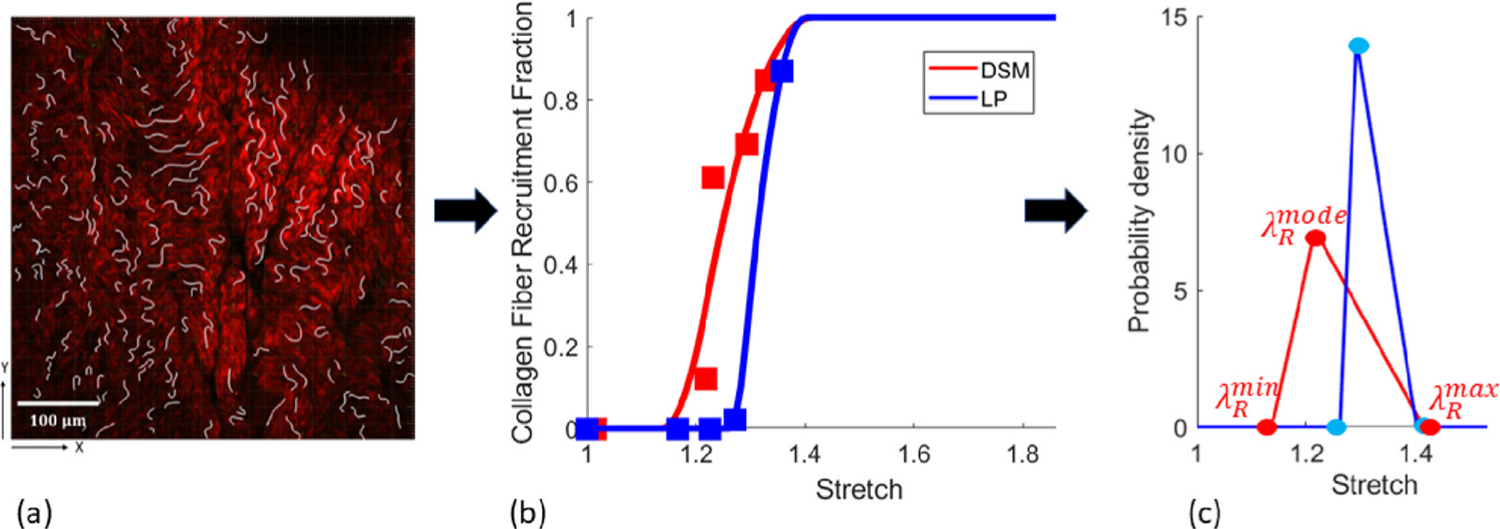
Procedure for fitting the fiber recruitment distribution function shown for representative data set: (a) Fiber tracing overlaid on multiphoton image (collagen fibers in red) (b) Fiber recruitment fraction versus stretch. (c) Fitted triangular recruitment distributions for each layer; $\lambda_{R}^{\text{min}}$, $\lambda_{R}^{\text{mode}}$ and $\lambda_{R}^{\text{max}}$ depicted for the detrusor layer.

The collagen fiber recruitment fraction was quantified in the wall layers at multiple loading levels following a previously established method (Cheng et al., 2018) and used to obtain fiber recruitment distribution functions (Cheng, 2022), see Fig. 3. Briefly, multiphoton image stacks were used to obtain 3D reconstructions of the collagen fiber architecture that were mapped to the corresponding stretch on the loading curve. Collagen fibers were traced in the stack of 2D slices corresponding to the depth of the 3D reconstruction (Filament function in Imaris, Bitplane, Switzerland) (Cheng et al., 2018). The fiber arc length was determined for each fiber tracing and the cord length was defined as the length of the best linear fit line to the same segment. Fiber straightness was defined as the ratio of chord length to arc length and a fiber was designated as recruited to load-bearing when its straightness reached 0.98 (Hill et al., 2012). The robustness of the fiber straightness measurement was validated from the residual of average fiber straightness (Cheng et al., 2018). The fraction of fibers recruited to load bearing was measured and the recruitment distribution (measured using MPM) is represented with a triangular probability density function *ρ*_*R*_ (Aparício et al., 2016); $\lambda_{R}^{q}$ relates to the minimum (*q* = *min*), modal (*q* = *mode*) and maximum (*q* = *max*) recruitment stretches of the distribution (see Fig. 3). More specifically:

(1) $$\rho_{R}\left({\bar{\lambda }}_{R}\right)=\{\begin{array}{ll} 0, & {\bar{\lambda }}_{R}\leq {\bar{\lambda }}_{R}^{\text{min}}\\ \frac{2\left({\bar{\lambda }}_{R}-{\bar{\lambda }}_{R}^{\text{min}}\right)}{{\bar{\lambda }}_{R}^{\text{width}}\left({\bar{\lambda }}_{R}^{\text{mode}}-{\bar{\lambda }}_{R}^{\text{min}}\right)}, & {\bar{\lambda }}_{R}^{\text{min}}<{\bar{\lambda }}_{R}\leq \lambda_{R}^{\text{mode}}\\ \frac{2\left({\bar{\lambda }}_{R}^{\text{max}}-{\bar{\lambda }}_{R}\right)}{{\bar{\lambda }}_{R}^{\text{width}}\left({\bar{\lambda }}_{R}^{\text{max}}-{\bar{\lambda }}_{R}^{\text{mode}}\right)}, & {\bar{\lambda }}_{R}^{\text{mode}}<{\bar{\lambda }}_{R}\leq {\bar{\lambda }}_{R}^{\text{max}}\\ 0, & {\bar{\lambda }}_{R}^{\text{max}}<{\bar{\lambda }}_{R}. \end{array}$$

where $\bar{\lambda }$ is used to signify the stretch measured relative to the reference configuration for the biaxial testing and multi-photon microscopy. The width of the fiber recruitment distribution is ${\bar{\lambda }}_{R}^{\text{width}}={\bar{\lambda }}_{R}^{max}-{\bar{\lambda }}_{R}^{min}$ and the skew ${\bar{\lambda }}_{R}^{\text{skew}}\in [0,1]$ is calculated as ${\bar{\lambda }}_{R}^{\text{skew}}=\left({\bar{\lambda }}_{R}^{\text{mode}}-{\bar{\lambda }}_{R}^{min}\right)/{\bar{\lambda }}_{R}^{\text{width}}$; ${\bar{\lambda }}_{R}^{\text{skew}}=0.5$ represents a symmetric distribution.

#### 2.2.4. Void stretch

For each tissue sample, we estimated the magnitude of biaxial stretch ${\bar{\lambda }}_{F}$ that corresponds to the stretch in the voiding configuration. This enables us to infer the collagen fiber stretch distribution at the onset of voiding (from the measured recruitment stretch distribution) which we use to inform the homeostatic fiber stretch distribution (Section 3.3). To determine ${\bar{\lambda }}_{F}$, we estimate the passive wall stress $\sigma_{\text{pass}}^{\text{void}}$ of the intact bladders at the onset of voiding. The method is as follows: for each bladder, we compute an equivalent unloaded spherical geometry, with volume equal to the measured unloaded volume, *V*_0_; we assume filled bladders have spherical geometries and use their filled volume to estimate their filled radii; we compute the voiding stretch $\lambda_{F}^{\left(0\right)}$ which is the ratio of the radii of the filled/unloaded spherical bladders. Using the Law of Laplace (equilibrium for a pressurized spherical membrane), we calculate the passive wall stress for the filled bladder $\sigma_{\text{pass}}^{\text{void}}$ for each case. The void stretch ${\bar{\lambda }}_{F}$ is then inferred directly from the biaxial stress–stretch data.

### 2.3. In silico analysis: Constrained mixture model

The CMMG model utilizes two timescales. Micturition occurs over a short time scale on the order of seconds. The variable *t* will be used when calculating metrics associated with the voiding cycle. In contrast, the bladder remodels to restore voiding function over weeks. Therefore, a second time variable *τ* is introduced for modeling the G&R process that returns voiding metrics to homeostatic values.

**Fig. 4.**
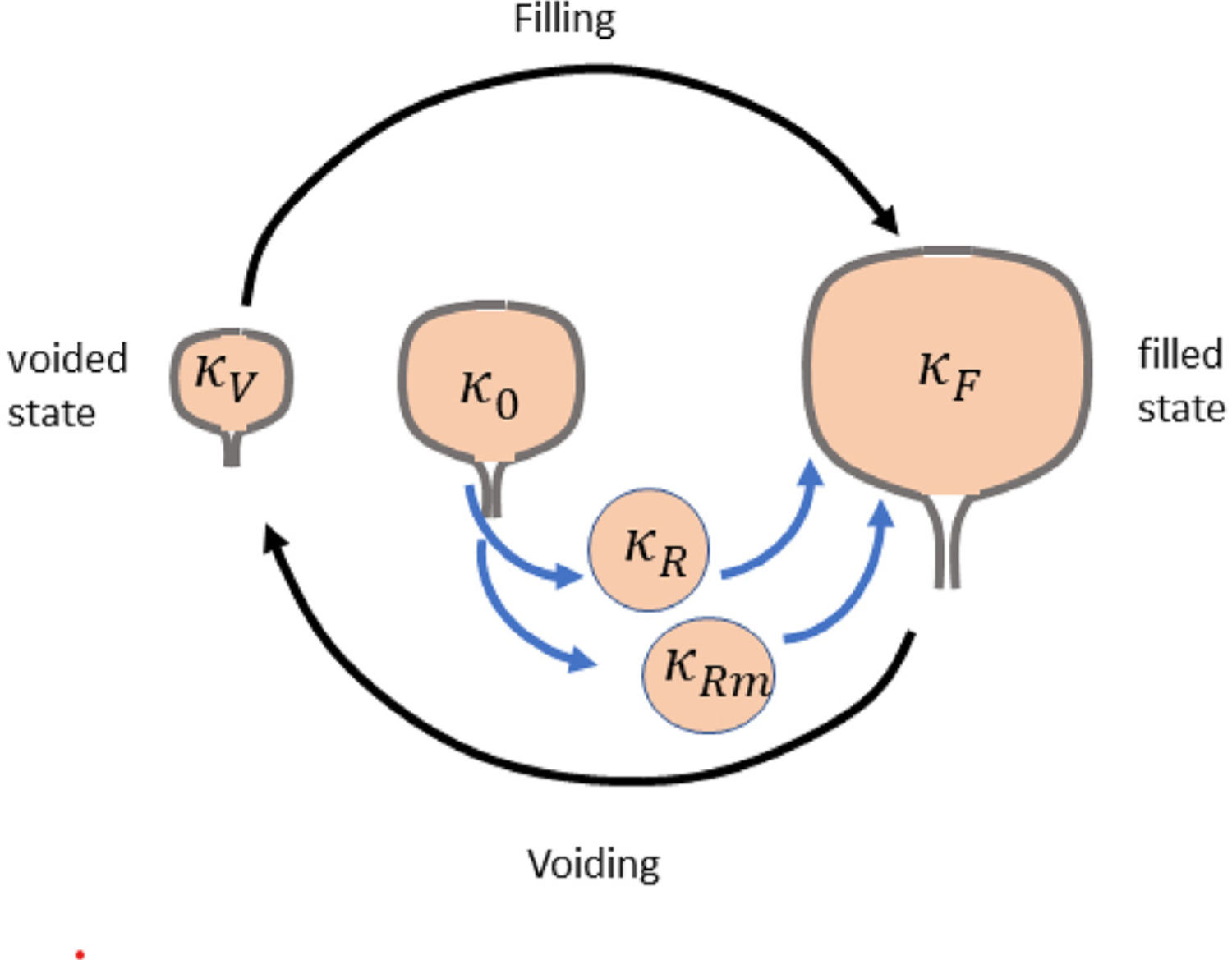
Configurations of the bladder during the voiding cycle. The collective (passive) response of the non-collagenous constituents is defined relative to the unloaded configuration of the bladder *κ*_0_. The passive mechanical response of collagen fibers and the active response of SMCs are defined relative to their natural reference configurations *κ*_*R*_ and *κ*_*Rm*_, respectively.

The bladder wall experiences large displacements that are modeled as quasi-static and constrained to be isochoric (as the bladder tissue is idealized as incompressible). We denote the filled configuration as *κ*_*F*_ the voided configuration as *κ*_*V*_ and the unloaded configuration as *κ*_0_. Additional, natural reference configurations (which can evolve) for the collagen fibers and SMCs are denoted as *κ*_*R*_ and *κ*_*Rm*_, respectively (Fig. 4). Here we introduce a general framework which accounts for the collagen and SMC orientations.

The kinematics for the deforming bladder are described in terms of the right Cauchy Green tensor $\underline{\underline{C}}={\underline{\underline{F}}}^{T}\underline{\underline{F}}$ where $\underline{\underline{F}}$ is the deformation gradient tensor. The tensor invariants for $\underline{\underline{C}}$ are $I_{1}=\text{tr}(\vec{C}),I_{2}=\left(\text{tr}{\left(\underline{\underline{C}}\right)}^{2}-\text{tr}\left({\underline{\underline{C}}}^{2}\right)\right)/2$ and $I_{3}=\text{det}(\underline{\underline{C}})=1$. The direction of the collagen fibers in the unloaded reference configuration is denoted by the unit vector ${\underline{a}}^{i}$ where *i* ranges over the number of fiber orientations for the given point within the tissue. The stretch $\lambda_{4}^{i}$ in the fiber direction is

(2) $${\left(\lambda_{4}^{i}\right)}^{2}=I_{4}^{i}={\underline{a}}^{i}\cdot \underline{\underline{C}}{\underline{a}}^{i}$$

i.e., associated with $I_{4}^{i}$, a pseudo-invariant of $\underline{\underline{C}}$ and ${\underline{a}}^{i}$. Due to the waviness of the collagen fibers, the (true) fiber stretch $\lambda_{c}^{i}$ is given by

(3) $$\lambda_{c}^{i}=\frac{\lambda_{4}^{i}}{\lambda_{Rc}^{i}}$$

where $\lambda_{Rc}^{i}$ denotes the collagen fiber recruitment stretch.

Similarly, denoting the SMC direction in the unloaded reference configuration by the unit vector ${\underline{a}}_{m}$, the stretch *λ*_4*m*_ in the SMC direction is

(4) $${\left(\lambda_{4m}\right)}^{2}=I_{4m}={\underline{a}}_{m}\cdot \underline{\underline{C}}{\underline{a}}_{m}$$

The (true) SMC stretch *λ*_*m*_ is related to the tissue stretch by

(5) $$\lambda_{m}=\frac{\lambda_{4m}}{\lambda_{Rm}}$$

where *λ*_*Rm*_ is the SMC recruitment stretch.

We assume an additive decomposition of the stresses arising from the passive and active components. For each layer, the passive contributions arise from collagen fibers $\left({\underline{\underline{\sigma }}}_{L,c}\right)$ as well as non-collagenous, isotropic component $\left({\underline{\underline{\sigma }}}_{L,nc}\right)$ that accounts for the collective (passive) response of elastin, amorphous matrix and low stress behavior of smooth muscle cells (Tuttle et al., 2021). The subscript *L* denotes the wall layer, *L = LP*, *DL* and *AD* denotes lamina propria, detrusor muscle layer and adventitial layer, respectively. At any point in the remodeling process, these passive components are assumed to be hyperelastic, with strain energy functions defined below. Finally, the smooth muscle tissue contributes an active component $\left({\underline{\underline{\sigma }}}_{a}\right)$ in the detrusor smooth muscle layer. The Cauchy stress in layer L due to the collective response of these components is

(6) $${\underline{\underline{\sigma }}}_{L}={\underline{\underline{\sigma }}}_{L,nc}+{\underline{\underline{\sigma }}}_{L,c}+{\underline{\underline{\sigma }}}_{L,a},\;L=LP,DL,AD.$$

where the active component is only non-zero in the DL. Hereafter, we will drop the layer designation and write ${\underline{\underline{\sigma }}}_{a}$.

#### 2.3.1. Strain energy functions for passive components

We introduce a strain energy function per unit reference volume for each layer. The strain energy function for the non-collagenous isotropic component (passive) is assumed to depend on the first invariant of $\underline{\underline{C}}$ while the collagen contribution arises from the collective contribution of recruited fibers. The response at the fiber level depends on the actual fiber stretch. Assuming an affine deformation, the collective response can be represented in terms of the tissue stretch in the fiber direction,

(7) $$\Psi_{L}=\Psi_{L,nc}\left(I_{1}\right)+\underset{i}{\sum }\Psi_{L,c}^{i}\left(I_{4}^{i}\right).$$

where *i* ranges over the number of fiber orientations (sometimes referred to as fiber families) accounted for in the model.

##### Isotropic constituents

The isotropic (non-collagenous) components are modeled as a neo-Hookean material (Tuttle et al., 2021), with strain energy function and corresponding Cauchy stress,

(8) $$\Psi_{L,nc}=k_{L,nc}\left(I_{1}-3\right),\;{\underline{\underline{\sigma }}}_{L,e}=2k_{L,nc}\underline{\underline{B}}$$

where *k*_*L*,*nc*_ being stiffness-like material constants, $\underline{\underline{B}}={\underline{\underline{FF}}}^{T}$ is the left Cauchy–Green deformation tensor.

##### Collagenous constituents

The constitutive model for the collagen accounts for the distribution of fiber recruitment (Lanir, 1983; Hill et al., 2012). The strain energy of the fiber ensemble is given by the convolution of the fiber strain energy $\left({\tilde{\Psi }}_{c}^{i}\right)$ and the recruitment distribution function $\left(\rho_{R}^{i}\right)$,

(9) $$\Psi_{c}\left(I_{4}^{i}\right)=m_{c}^{i}\cdot \underset{i}{\sum }\int_{1}^{\sqrt{I_{4}^{i}}}{\tilde{\Psi }}_{c}^{i}\left(\lambda_{c}^{i}\right)\rho_{R}^{i}\left(\lambda_{Rc}^{i}\right)d\lambda_{Rc}^{i}.$$

where $m_{c}^{i}$ is the (dimensionless) normalized mass density of collagen fibers that can adapt to simulate collagen growth/atrophy (Watton et al., 2004). The strain energy for each fiber is taken to be quadratic (Chen, 2014; Aparício et al., 2016; Zhang et al., 2016) such that it yields a linear force–stretch relation: $\left(\lambda_{c}^{i}\right)$

(10) $${\tilde{\Psi }}_{c}^{i}\left(\lambda_{c}^{i}\right)=\{\begin{array}{ll} \frac{k_{c}^{i}}{2}\cdot {\left(\lambda_{c}^{i}-1\right)}^{2} & \lambda_{c}^{i}\geq 1\\ 0, & \text{ otherwise ,} \end{array}$$

where *k*_*c*_ is a stiffness-like material constant. Using triangular recruitment distribution functions (1) yields analytic expressions for the strain energy from which expressions for the collagen stress (which are continuously differentiable, *C*^1^) can be derived (e.g. see Chen (2014), Bhogal et al. (2019)). This facilitates an efficient numerical implementation and remodeling scheme. Alternate distributions could also be used (Hill et al., 2012).

#### 2.3.2. Constitutive model for active SMC response

The active stress generated by the bladder during voiding is correlated to nerve activity (Le Feber et al., 1997) and occurs over a large range of bladder contractility (Uvelius, 2001). We define the active (Cauchy) stress in the current direction of the SMC, $\sigma_{m}^{\text{act}}$, to be a function of SMC stretch (*λ*_*m*_), nervous stimuli *S*(*t*) and normalized SMC mass-density *m*_*m*_:

(11) $$\sigma_{m}^{\text{act}}=\{\begin{array}{ll} 0 & \lambda_{m}\leq \lambda_{m}^{\text{min}}\\ S(t)m_{m}k_{m}^{\text{act}}g_{m}\left(\lambda_{m}\right) & \lambda_{m}^{\text{min}}\leq \lambda_{m}\leq \lambda_{m}^{\text{max}}\\ 0 & \lambda_{m}\geq \lambda_{m}^{\text{max}} \end{array}$$

where *g*_*m*_(*λ*_*m*_) is a *C*^1^ (continuous function that satisfies *g*_*m*_(*λ*_*m*_) ≥ 0, $g_{m}\left(\lambda_{m}^{\text{min}}\right)=g_{m}\left(\lambda_{m}^{\text{max}}\right)=0$ and has one maximum on the interval $\left[\lambda_{m}^{\text{min}},\lambda_{m}^{\text{max}}\right]$. The specific functional form we use is detailed in Section 2.6.

The stimuli *S*(*t*) is chosen so that it ramps up at the onset of voiding and incorporates a (urethral) feedback mechanism to decrease *S*(*t*) to zero when the flow rate reduces to a (relatively) low magnitude, $Q_{\text{crit}}^{S}$ near the end of voiding at *t* = *t*_*crit*_, i.e.

(12) $$S(t)=\{\begin{array}{ll} 1-\frac{1}{1+{\left(t/k_{m1}\right)}^{4}} & 0\leq t\leq t_{\text{crit}}\\ \left(1-\frac{1}{1+{\left(t_{\text{crit}}/k_{m1}\right)}^{4}}\right)\left(\frac{1}{1+{\left(\left(t-t_{\text{crit}}\right)/k_{m2}\right)}^{4}}\right) & t_{\text{crit}}<t\leq t_{\text{end}}\\ 0 & t>t_{\text{end}} \end{array}$$

### 2.4. In silico: Micturition model

In a healthy bladder, voiding (micturition) commences when contraction of the SMCS in the bladder wall is sufficient to elevate the internal bladder pressure above the *cut off pressure*, *P*_*c*_. Immediately after BOO inducing surgery, rats exhibit overflow incontinence due to an inability of the BOO bladders to generate sufficient pressures to overcome the additional urethral resistance imposed by the partial obstruction. Namely, during a post surgery period, regular voiding and filling cycles are not achievable and urine only exits the bladder through leakage. The leakage is driven by elevated internal pressure due to the passive response of the wall and a steady state is achieved where outflow matches inflow. As the BOO bladder remodels in response to obstruction, it recovers voiding functionality. These two distinct modes of micturition in the BOO bladder will be referred to as the *leakage mode* and the *functional mode*, respectively. In contrast, a healthy bladder only displays the functional mode. The modeling strategy for these two modes is elaborated on below.

#### Functional mode.

In this state, the SMCs are able to generate sufficient active wall tension to induce voiding. For the *in silico* model, this process is simulated through a coupling between SMC stretch and the active stress generated by the SMCs. As the bladder fills and enlarges, the nervous stimulus function *S*(*t*) is triggered when the SMC stretch reaches a target value. Subsequently, SMC active stress increases and flow is initiated when the internal bladder pressure (*passive + active*) exceeds the cutoff pressure (*p*_*c*_). The bladder continues to contract until the pressure falls below the cutoff pressure and voiding stops. Following voiding, the stimulus function is set to zero, the bladder begins filling again and the cycle repeats.

The temporal dynamics of the pressure and outflow-rate of the active bladder are computed during voiding. For both modes we assume that the bladder has a constant filling rate, *Q*_*in*_. Following earlier works (Griffiths, 1973; Bates et al., 1975), we assume a linear relationship between voiding flow rate *Q*(*t*) and bladder pressure *P* (*λ*, *t*):

(13) $$Q(t)=\{\begin{array}{ll} \frac{1}{\alpha }\left(P(\lambda ,t)-P_{c}\right) & P(\lambda ,t)>P_{c}\\ 0 & P(\lambda ,t)\leq P_{c} \end{array}$$

where *α* is the slope of the Pressure-Flow relationship and is a measure of urethral resistance. As voiding progresses, the updated volume is computed to calculate updated pressure (*P* (*λ*, *t*)). On completion of voiding, relevant metrics are calculated (volume voided, residual volume, voiding duration, contractile range).

#### Leakage mode.

In leakage model, the flow rate matches the filling rate of the bladder, i.e. *Q*_*out*_ = *Q*_*in*_. The passive pressure (*P*^*leaky*^) corresponding to this steady state condition can be determined from Eq. (13), i.e.

(14) $$P^{\text{leaky}}=\alpha Q_{in}+P_{c}^{BOO}$$

This is used to determine the deformation of the bladder whilst in the leaky state.

### 2.5. In silico: Homeostasis, growth and remodeling

In this section we provide an overview of the strategy for modeling growth and remodeling in the bladder wall. The specifics for obtaining material parameters are given in later sections as part of the implementation of the CMMG model.

#### 2.5.1. Homeostasis

##### Collagen.

As elaborated on below, motivated by earlier experimental data for collagen fibers (Cheng et al., 2018) as well as results obtained in the present work, see Section 3.3, we assume collagen acts as a protective sheath. Moreover, we assume collagen fibers are deposited with a homeostatic stretch distribution about the onset of voiding. The three parameters needed for the homeostatic stretch distribution at homeostasis are $\lambda_{c,h}^{i,\text{min}}$, $\lambda_{c,h}^{i,\text{mode}}$ and $\lambda_{c,h}^{i,\text{max}}$. As detailed in Section 3.3 these values are inferred from the biaxial experiments.

##### SMC.

We assume SMCs configure with a preferred homeostatic stretch at the onset of voiding, denoted *λ*_*m*,*h*_. The magnitude of *λ*_*m*,*h*_ must be sufficiently greater than $\lambda_{m}^{\text{min}}$ so that the SMC can generate active stress over a desired contractile range. In addition, we hypothesize that the SMC is configured to the left of the maximum of the active pressure–SMC stretch relationship so that the (maximum) active pressure monotonically decreasing as the bladder voids.

#### 2.5.2. Remodeling

##### Collagen.

We capture the effect of fiber deposition and degradation in altered configurations (Humphrey, 1999) by remodeling the reference configuration at which fibers are recruited to load bearing (Watton et al., 2004) so that the fiber stretch distributions remodel towards a preferred homeostatic stretch distribution (Aparício et al., 2016) about the onset of voiding :

(15) $$\frac{\partial \lambda_{Rc}^{i,\text{min}}(X,\tau )}{\partial \tau }=\alpha_{c}\left(\frac{{\lambda_{c}^{i,\text{max}}(X,\tau )|}_{\kappa_{\text{max}}}-\lambda_{c,h}^{i,\text{max}}}{\lambda_{c,h}^{i,\text{max}}}\right)$$

(16) $$\frac{\partial \lambda_{Rc}^{i,\text{mod}}(X,\tau )}{\partial \tau }=\alpha_{c}\left(\frac{{\lambda_{c}^{i,\text{mod}}(X,\tau )|}_{\kappa_{\text{max}}}-\lambda_{c,h}^{i,\text{mod}}}{\lambda_{c,h}^{i,\text{mod}}}\right)$$

(17) $$\frac{\partial \lambda_{Rc}^{i,\text{max}}(X,\tau )}{\partial \tau }=\alpha_{c}\left(\frac{{\lambda_{c}^{i,\text{min}}(X,\tau )|}_{\kappa_{\text{max}}}-\lambda_{c,h}^{i,\text{min}}}{\lambda_{c,h}^{\text{min}}}\right)$$

where *α*_*c*_ is a remodeling rate parameter and the max, mode and minimum collagen fiber stretches evaluated at *κ*_*F*_ are, respectively:

(18) $${\lambda_{c}^{i,max}|}_{\kappa_{F}}=\frac{{\lambda_{4}^{i}|}_{\kappa_{F}}}{\lambda_{Rc}^{i,min}},{\;\lambda_{c}^{i,mod}|}_{\kappa_{F}}=\frac{{\lambda_{4}^{i}|}_{\kappa_{F}}}{\lambda_{Rc}^{i,mod}},\text{ and }{\;\lambda_{c}^{i,min}|}_{\kappa_{F}}=\frac{{\lambda_{4}^{i}|}_{\kappa_{F}}}{\lambda_{Rc}^{i,max}}\text{.}$$

##### SMC.

We hypothesize that SMCs remodel to maintain their stretch towards a homeostatic value (*λ*_*c*,*h*_) about the onset of voiding:

(19) $$\frac{\partial \lambda_{Rm}(X,\tau )}{\partial \tau }=\alpha_{m}\left(\frac{{\lambda_{m}(X,\tau )|}_{\kappa_{F}}-\lambda_{m,h}}{\lambda_{m,h}}\right)$$

where ${\lambda_{m}|}_{\kappa_{F}}$ is the SMC stretch at onset of voiding, *λ*_*Rm*_ is the SMC recruitment stretch, *λ*_*m*,*h*_ is the SMC homeostatic stretch and *α*_*m*_ is a remodeling rate parameter.

#### 2.5.3. Growth

The bladder responds to outlet obstruction with SMC hypertrophy so that it can generate sufficient force to overcome the outlet resistance and successfully void. We simulate the mechanical consequence of SMC hypertrophy by evolving the SMC mass density. The regulatory homeostatic setpoints that drive SMC hypertrophy are not known for the bladder. We propose and explore possible candidates that act to maintain bladder voiding functionality, i.e. SMC growth acts to restore: (i) volume voided (*GF*_1_); (ii) average voiding flow rate (*GF*_2_); (iii) contractile range (*GF*_3_).

##### Muscle growth driven by total voided volume.

In this proposed mechanism, muscle grows to maintain the total volume voided for the bladder,

(20) $$\frac{\partial m_{smc}}{\partial \tau }=\beta_{smc}m_{smc}\left(\frac{V_{\text{void}}^{0}-V_{\text{void}}(\tau )}{V_{\text{void}}^{0}}\right)$$

where $P_{\text{void}}^{\text{0}}$ is the volume voided at *τ* = 0 (normal bladder) and *V*_*void*_ (*τ*) is the volume voided at time *τ*; *β*_*smc*_ is the growth rate parameter of SMC.

##### Muscle growth driven by average flow rate during voiding.

For this proposed mechanism, we suppose there is a feedback mechanism to drive SMC growth to restore the average voiding flow rate. In principal, this requires the urethra to sense flow rate (Birder et al., 2014). Specifically,

(21) $$\frac{\partial m_{smc}}{\partial \tau }=\beta_{smc}m_{smc}\left(\frac{Q_{avg}^{0}-Q_{avg}(\tau )}{Q_{avg}^{0}}\right)$$

where *Q*_*avg*_(0) is the average voiding flow rate at *t* = 0 (sham bladder) and *Q*_*avg*_ (*τ*) is the average voiding flow rate at time *τ*.

##### Muscle growth driven by muscle contraction range during voiding.

For this proposed mechanism, muscle grows to maintain the contraction range *λ*_*m*−*cyc*_ during voiding, which is defined as the difference between the muscle stretch at the filled state $\lambda_{m}^{F}$ and the muscle stretch at the voided state $\lambda_{m}^{V}$, i.e.

(22) $$\frac{\partial m_{smc}}{\partial \tau }=\beta_{smc}m_{smc}\left(\frac{\lambda_{m-cyc}^{0}-\lambda_{m-cyc}(\tau )}{\lambda_{m-cyc}^{0}}\right)$$

where $\lambda_{m-cyc}=\lambda_{m}^{F}-\lambda_{m}^{V}$ and, $\lambda_{m-cyc}^{0}$ and *λ*_*m*−*cyc*_(*τ*), are the muscle contractile ranges during voiding at *τ* = 0 and *τ*, respectively.

### 2.6. In silico : Formulation of the CMMG model for an idealized spherical membrane

**Fig. 5.**
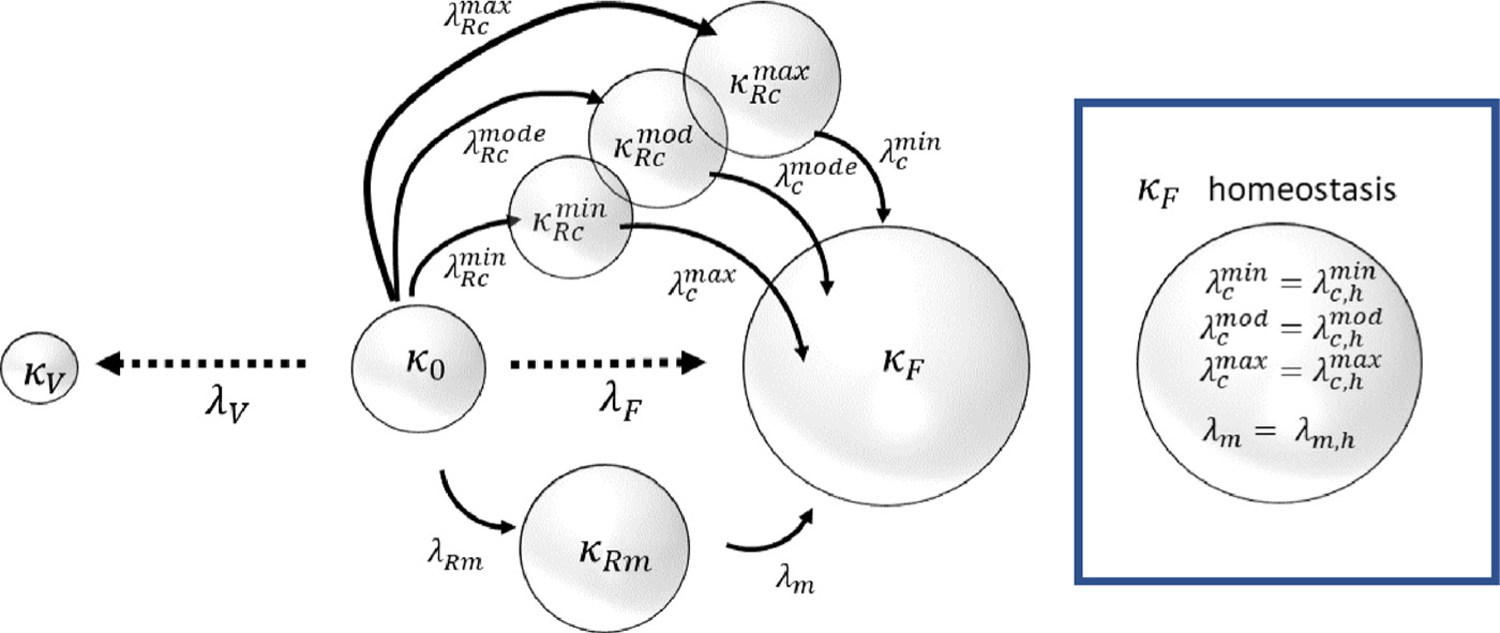
Schematic of different states of the CMMG model with spherical geometry. *κ*_0_, *κ*_*F*_ and *κ*_*V*_ represent the unloaded, filled and voided states of the bladder, respectively. *κ*_*Rm*_ and *κ*_*Rc*_ are intermediate recruitment configurations for SMC and collagen fibers in which the stretch of the constituent/cell is 1. Collagen fibers have a distribution of recruitment, those with the lowest value of recruitment achieve the highest stretches in the voiding configuration *κ*_*F*_. Conversely the last fibers of the distribution to be recruited $\left(\lambda_{Rc}^{\text{max}}\right)$ will have the lowest stretches $\left(\lambda_{c}^{\text{min}}\right)$. At homeostasis, in the voiding configuration, the collagen fiber stretch distribution coincides with the homeostatic distribution and the SMC stretch also takes its homeostatic value.

In this section, we formulate the equations needed to apply the general theoretical developments of the previous sections to an idealized spherical membrane geometry (see Fig. 5). The contributions of each of the wall components will be treated as isotropic. This yields analytic solutions that can be used to efficiently explore the bladder behavior (on both long and short time scales) using the CMMG model. The various reference configurations needed to model the filling/voiding cycle as well as the unloaded configuration of the bladder are shown in Fig. 5.

#### 2.6.1. Membrane model of bladder

The governing equation for quasi-static inflation of a spherical membrane composite is (Watton et al., 2009a):

(23) $$p=\frac{2H_{0}}{R_{0}\lambda^{3}}\left(\sigma_{nc}+r_{LP}^{H}\sigma_{c,LP}+r_{DL}^{H}\left(\sigma_{c,DL}+\sigma_{m}^{act}\right)+r_{AD}^{H}\sigma_{c,AD}\right)$$

where *p* is the transmural pressure, *R*_0_ the unloaded radius, *λ* is the tissue stretch (ratio of current and unloaded radii), $r_{L}^{H}$ denotes the ratio of the thickness of layer *L* to unloaded wall thickness *H*_0_ (*L* = *LP*, *DL*, *AD*), *σ*_*nc*_ denotes the Cauchy stress of the non-collagenous constituents, $\sigma_{c_{L}}$ denotes the Cauchy stress of collagen in layer *L* and $\sigma_{m}^{\text{act}}$ denotes the active SMC Cauchy stress, which only contributes to the DL layer.

For the active SMC stress constitutive equation, (11), we choose g_*m*_(*λ*_*m*_) to be a (simple) polynomial function which captures the active stress stretch response (Seydewitz et al., 2017) and yields a monotonically increasing active-pressure stretch relationship over the working range of SMC stretch during voiding (Peng et al., 2020),

(24) $$g_{m}\left(\lambda_{m}\right)=\left(\lambda_{m}^{4}+\lambda_{m}^{2}\right)\left(\lambda_{m}-\lambda_{m}^{min}\right)\left(\lambda_{m}^{max}-\lambda_{m}\right).$$

Insertion of Eqs. (5), (11) and (24) into (23) yields the contribution to the transmural pressure from the active SMC (*p*_*act*_):

(25) $$p_{\text{act}}\left(\lambda_{m}\right)=\frac{2H_{0}k_{m}^{\text{act}}}{R_{0}\lambda_{Rm}^{3}}S(t)m_{m}\left(\lambda_{m}+\left(1/\lambda_{m}\right)\right)\left(\lambda_{m}-\lambda_{m}^{min}\right)(\lambda_{m}^{max}-\lambda_{m}).$$

##### Evolution of wall thickness.

Hypertrophy of SMC will lead to a thickening of the bladder wall. The evolving wall thickness can be inferred from initial constituent volume fractions and evolution of the mass-densities; this allows for comparison of evolved thickness and stresses with experimental observations. For the spherical membrane model bladder, the evolved wall thickness in the voiding configuration, $h_{V}^{g}(\tau )$, is

(26) $$h_{V}^{g}(\tau )=\frac{\hat{\tau }(t)H_{0}}{\lambda_{V}^{2}}$$

where *λ*_*V*_ (*τ*) is the stretch at onset of voiding and the normalized volumetric growth $\hat{\tau }(t)$ is

(27) $$\hat{v}(\tau )=f_{e}^{0}m_{e}(\tau )+f_{c}^{0}m_{c}(\tau )+f_{m}^{0}m_{m}(\tau ),$$

where $f_{e}^{0}$, $f_{c}^{0}$, $f_{m}^{0}$ are the initial volume fractions of elastin, collagen and SMC which we take to be 0.01, 0.29, 0.70, respectively (Nagatomi et al., 2005; Toosi et al., 2008). Here we have not accounted for elastogenesis or collagen growth so take *m*_*e*_(*τ*) = *m*_*c*_(*τ*) = 1.

##### Comparison of model with biaxial mechanical data.

The growth model has a different unloaded reference configuration (*κ*_0_) to the reference configuration of the biaxial mechanical tests. To enable comparison of the stress–stretch response, we map the stretches so that the void stretches are consistent. Hence, to map the biaxial mechanical data relative to *κ*_0_, we multiply the biaxial stretch $\bar{\lambda }$ by $\lambda_{F}/{\bar{\lambda }}_{F}$. Conversely to map the simulation stretch *λ* to the biaxial configuration, we multiply by the stretch *λ* by ${\bar{\lambda }}_{F}/\lambda_{F}$.

## 3. Results: In vitro and In vivo studies

In this section, we provide the *in vivo* and *in vitro* results for BOO and sham bladders that inform the CMMG model of BOO. Constitutive model parameters for the bladder wall are calibrated to the *in vivo* and *in vitro* data for the sham bladder. The micturition as well as G&R modeling results for the BOO bladder are presented separately in Section 4.

### 3.1. In vivo cystometry measurements

Results for urodynamic metrics for sham/BOO bladders are detailed in Table 2. At 4 weeks post surgery, the remodeled BOO bladders all recovered functional voiding. The remodeled BOO bladders were capable of generating more than double the maximum voiding pressure to overcome the outlet obstruction. In contrast, the maximum filling pressure remained low at approximately 300 Pa.

We observed that whilst the dimensions of the unloaded bladder almost doubled, Table 3, the mean volume voided by sham and BOO bladders were almost equal and consequently the BOO bladder had a larger residual volume. Of note, the BOO bladder took significantly longer to void, i.e. an increase of almost fourfold.

### 3.2. In vitro bladder measurements

#### 3.2.1. Bladder dimensions

The BOO bladder increased significantly in size relative to the sham bladder with an average increase in diameter and height of 89% (P=0.0009) and 62% (P = 0.0045), respectively, Table 3. The wall thickness changed significantly in the BOO bladder, increasing from 0.78 to 1.56 mm (P = 0.01).

#### 3.2.2. In vitro measurements of collagen recruitment

The initiation of recruitment was significantly delayed in both layers of the BOO bladder relative to the sham, with a larger difference in the LP layer (see Table 4). For the BOO bladder, the average value of ${\bar{\lambda }}_{R}^{\text{min}}$ was 1.27 ± 0.02 compared with 1.17 ± 0.04 in the sham, (*P* = 0.03). Whereas, for the DP layer, the average value of ${\bar{\lambda }}_{R}^{\text{min}}$ was 1.13 ± 0.02 for BOO bladders compared with 1.08 ± 0.02 for sham (*P* = 0.03).

For both BOO and sham bladders, collagen fiber recruitment generally initiated in the DL prior to the LP (n=3/4 for BOO, 4/4 for sham). These differences were significant for both BOO (*P* = 0.0007) and sham bladders (*P* = 0.04).

The stretch at the onset of collagen recruitment was generally greater than the (computed) void stretch (n=3/4 BOO, n=3/4 sham). For cases where the recruitment initiated ahead of voiding, the differences were quite small (S1: 1.07 versus 1.06, B4: 1.08 versus 1.06). This suggests collagen plays little mechanical role during filling, i.e. it acts as a protective mechanical sheath against over-filling.

#### 3.2.3. In vitro results from biaxial testing

**Fig. 6.**
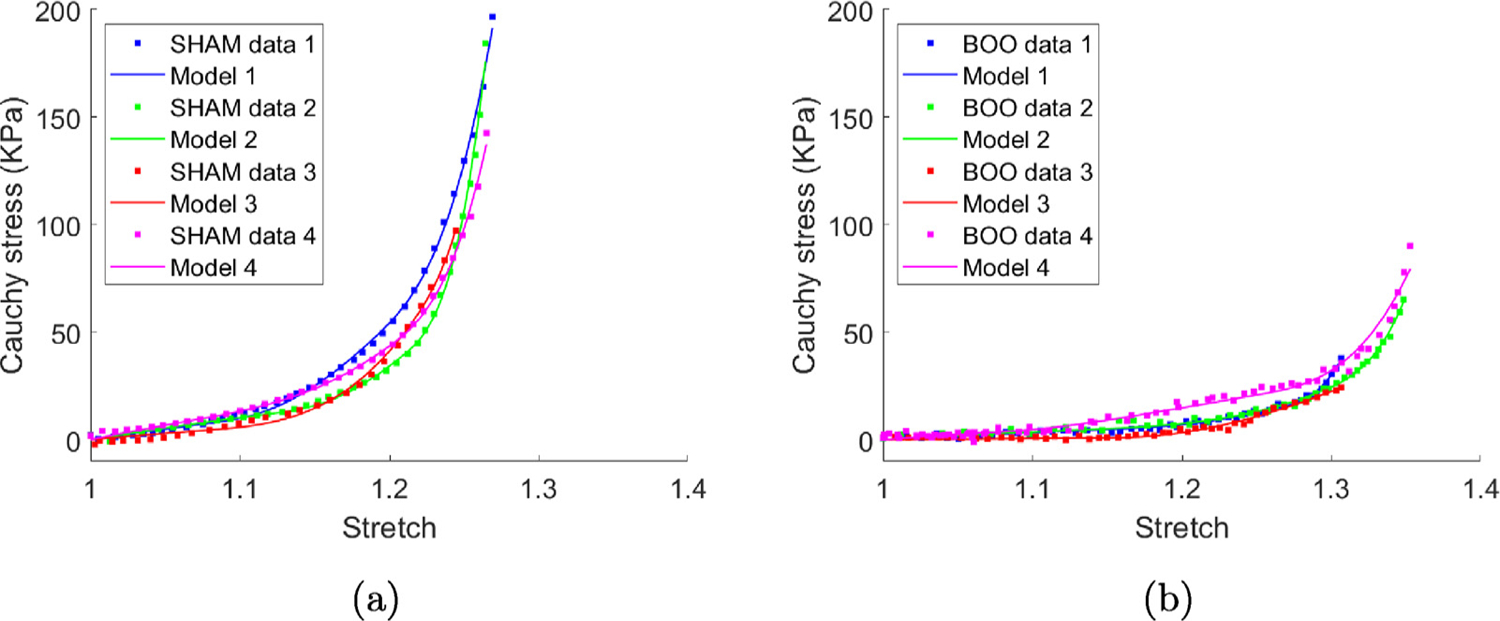
Constitutive model is fit to the (a) sham and (b) BOO biaxial data.

**Fig. A.20.**
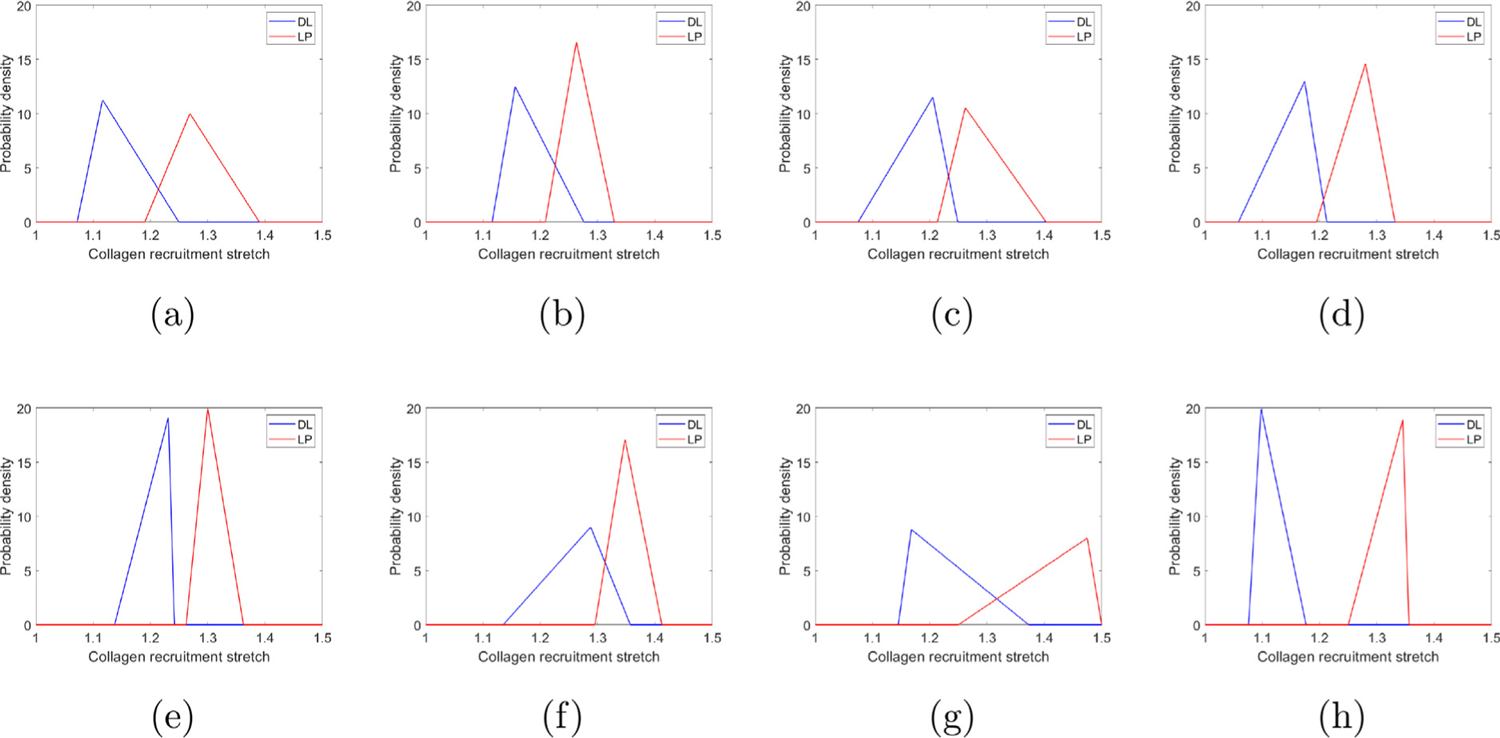
Fitted recruitment stretch distributions in the lamina propria and detrusor layer for sham (a-d) and BOO (e)–(h) bladders.

Both the biaxial stress–stretch data and the corresponding fit of the constitutive model for passive wall components are shown in Fig. 6 for all samples. The material parameters for the individual cases are provided in Appendix A, (Table A.7 and Fig. A.20). The initial soft toe region is well described by a Neo-Hookean model in all cases. With increasing stretch, the collagen fibers are recruited to load bearing in the DL followed by LP, resulting in a transition regime followed by a high stress regime (Cheng et al., 2018). The composite constitutive model is able to accurately capture the mechanical response for all sham and BOO tissue samples, Fig. 6.

**Fig. 7.**
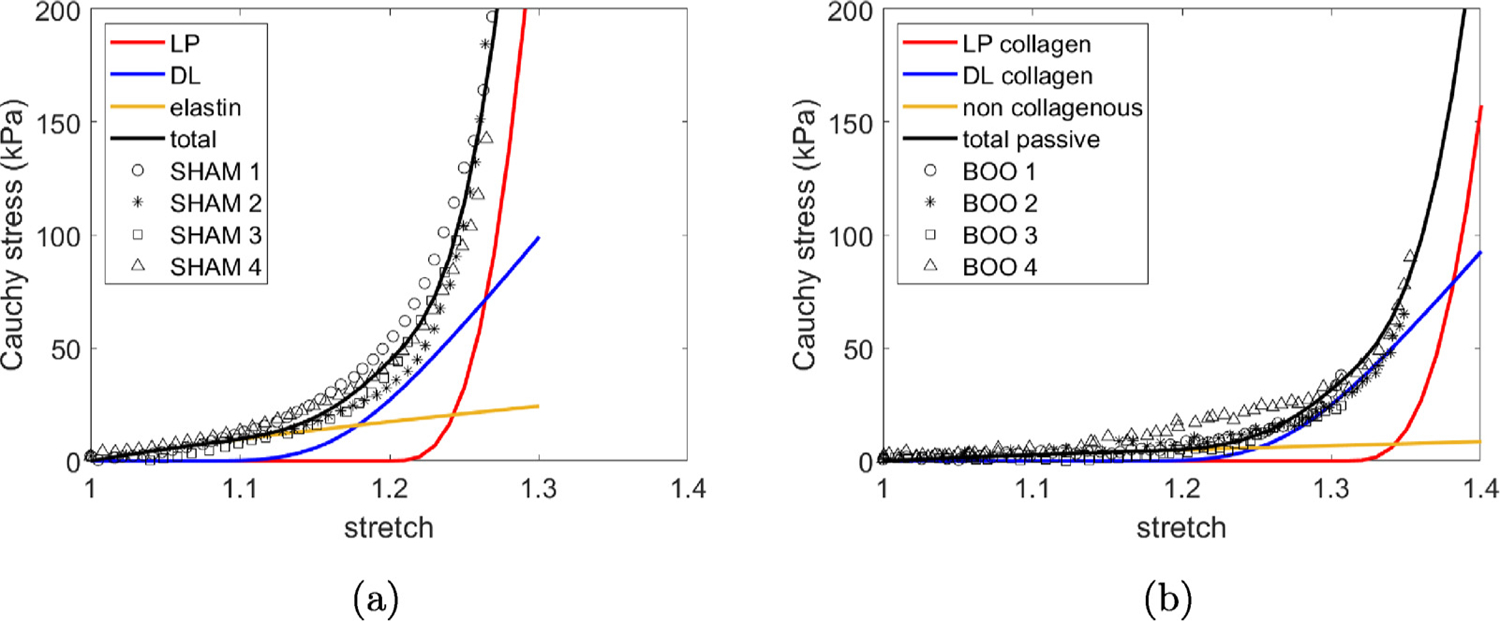
(a) Sham and (b) BOO models fit to experimental data which illustrate the contribution of individual components to the loading curves for the model.

The stress stretch curve is then redrawn in Fig. 7 with the distribution of stress across the wall layers explicitly shown. The delayed contribution of the DL and LP until after collagen recruitment is initiated in these layers is clear. As a consequence, the contribution to load bearing comes solely from the isotropic layer prior to the initiation of collagen recruitment in the DL.

### 3.3. Deposition stretches

**Fig. 8.**
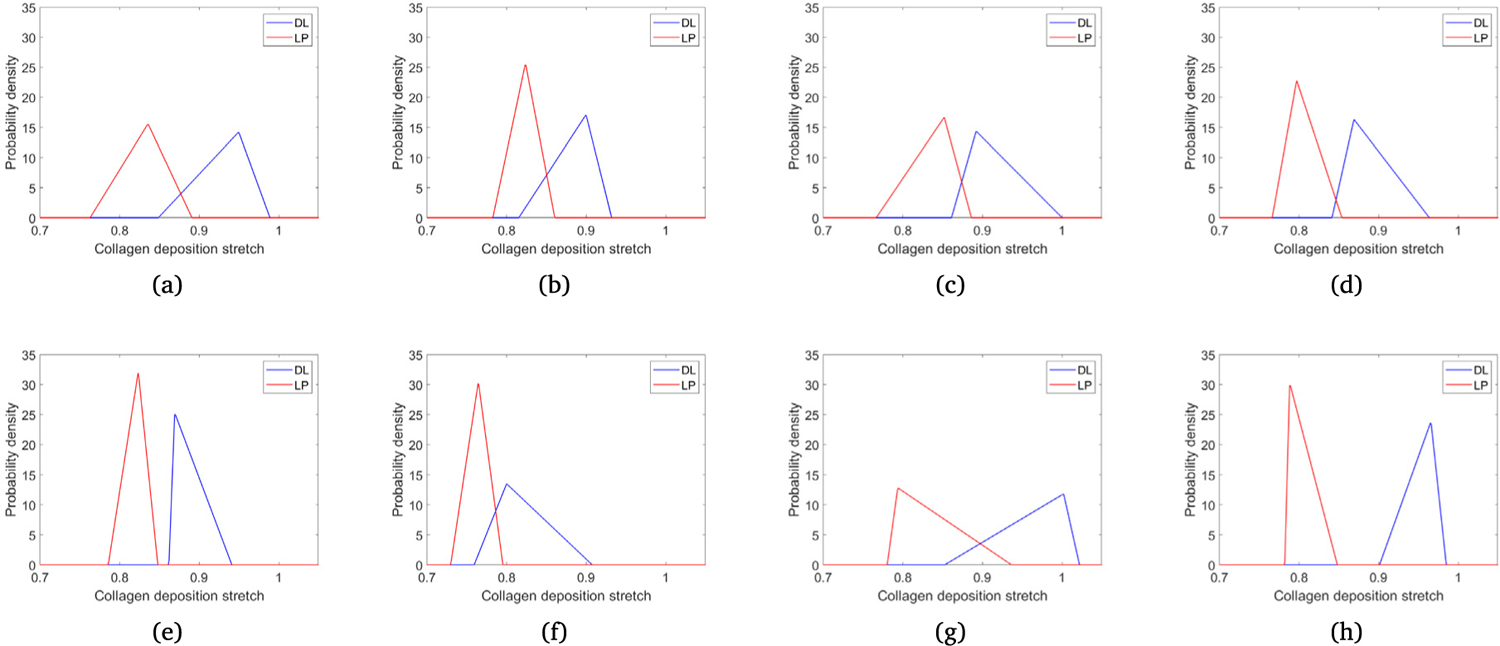
Collagen fibre deposition stretch distributions in the lamina propria and detrusor layer for sham (a-d) and BOO (e)–(h) bladders. These are inferred from recruitment stretch distributions and tissue stretch at onset of voiding.

The growth model of the bladder utilizes the assumption that collagen remodels to maintain a preferred homeostatic stretch distribution about the voiding configuration; we use the terminology *deposition stretch* for these homeostatic stretch values, denoted with a subscript *h*. The *deposition stretch* distributions for each sample (Fig. 8) are inferred from their recruitment stretch distributions and the (estimated) tissue stretch ${\bar{\lambda }}_{F}$ at the onset of voiding, Table 4, e.g. $\lambda_{c,h}^{\text{max}}={\bar{\lambda }}_{F}/{\bar{\lambda }}_{Rc}^{\text{min}}$. In all but 1 cases, the deposition stretch distribution is less than 1, i.e. LP and DL collagen are non-load bearing at the onset of voiding. Given that DL collagen recruits earlier, the DL deposition stretch distribution is greater than the LP deposition stretch distribution. Of significance, the mean sham LP and DL deposition distributions are of similar magnitudes to the mean BOO LP and DL deposition distributions, Table 5. This observation supports our hypothesis that the collagen remodels to maintain its stretch distribution towards a preferred homeostatic distribution about the onset of voiding.

## 4. Results: In silico studies

In this section, the CMMG model is applied to the idealized case of a spherical membrane composed of nonlinear isotropic components. Model predictions of the evolving properties of the bladder post partial obstruction surgery are compared with the experimental results at 4 weeks post-BOO. Lastly, the implications of the conjectured theories for homeostatic set points are contrasted to elucidate the most likely driver of SMC growth. Table 6 summarizes the CMMG model parameters used in these simulations; the methodology for determination of these parameters is detailed in Appendix B.

### 4.1. Modeling filling/voiding in the sham bladder

**Fig. 9.**
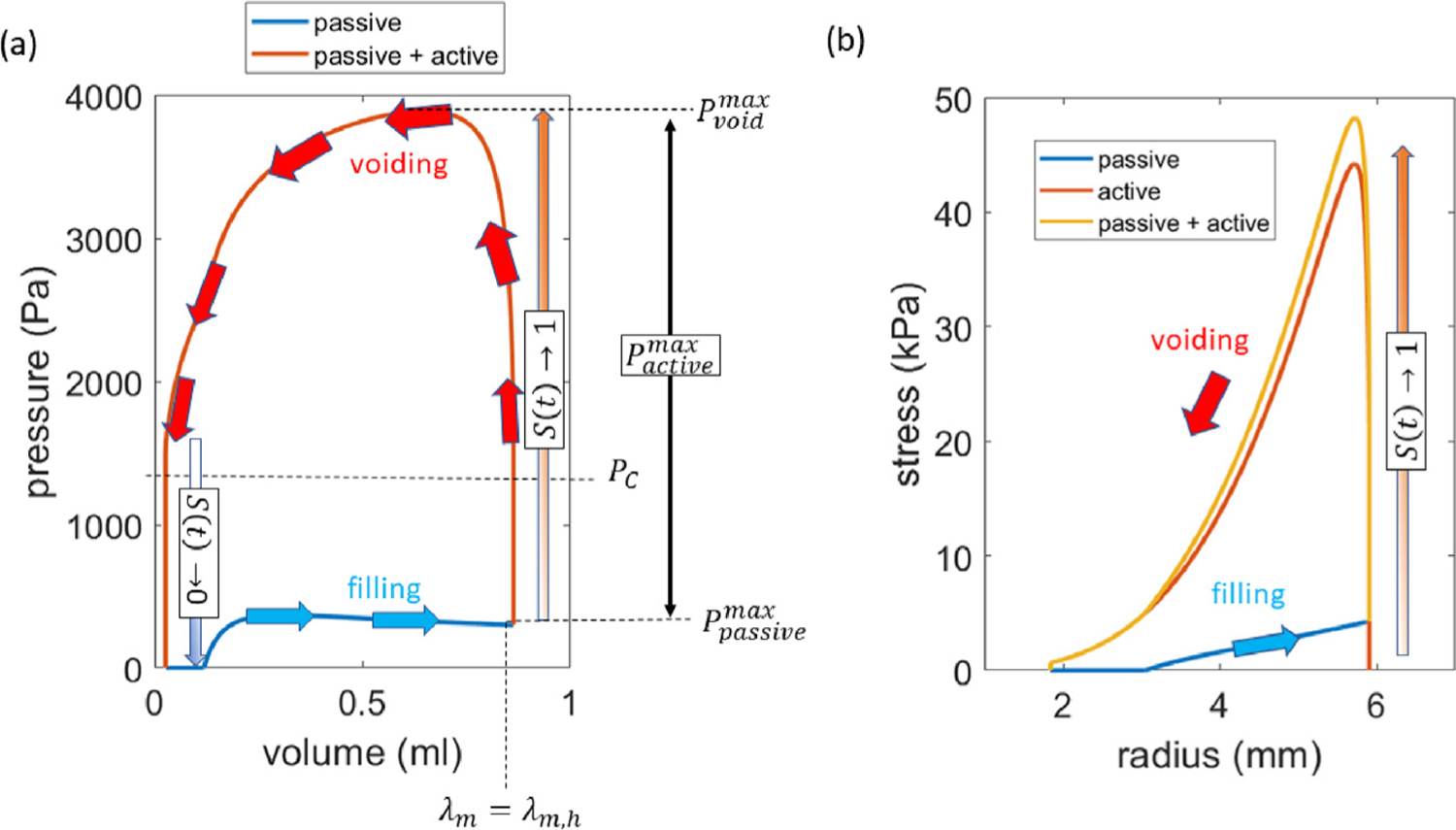
(a) Pressure–volume loop of the model of healthy (sham) bladder illustrating the filling and voiding cycle. (b) The corresponding Cauchy stress during filling and voiding, also for the sham bladder.

Essential features of the pressure–volume loop as predicted by the CMMG model are illustrated in Fig. 9a. The bladder fills under low pressure. As it enlarges, the SMC stretch increases, and when the SMC stretch equals the homeostatic stretch, i.e. *λ*_*m*_ = *λ*_*m*,*h*_, the bladder is triggered to void. Subsequently, the stimulus function *S*(*t*) increases and the bladder initially undergoes an isovolumetric contraction. When the bladder pressure exceeds the cut-off pressure (*P*_*c*_), the bladder begins to void. In the early stage of voiding, it can be seen that the bladder pressure continues to increase; this is a consequence of *S*(*t*) still increasing. Once *S*(*t*) = 1, the decrease in bladder volume is accompanied by a decrease in pressure, i.e. it follows the functional form of the active pressure–stretch relationship. Following cessation of flow, the active SMC stress reduces to zero (isovolumetric relaxation). The Cauchy stress during the filling-voiding cycle is depicted in Fig. 9(b). It can be seen that the passive stress increases linearly as the bladder fills and the passive component makes a small contribution to the pressure generated during voiding. The collagen is configured to be a protective sheath so does not contribute to the stress during filling/voiding cycle.

**Fig. 10.**
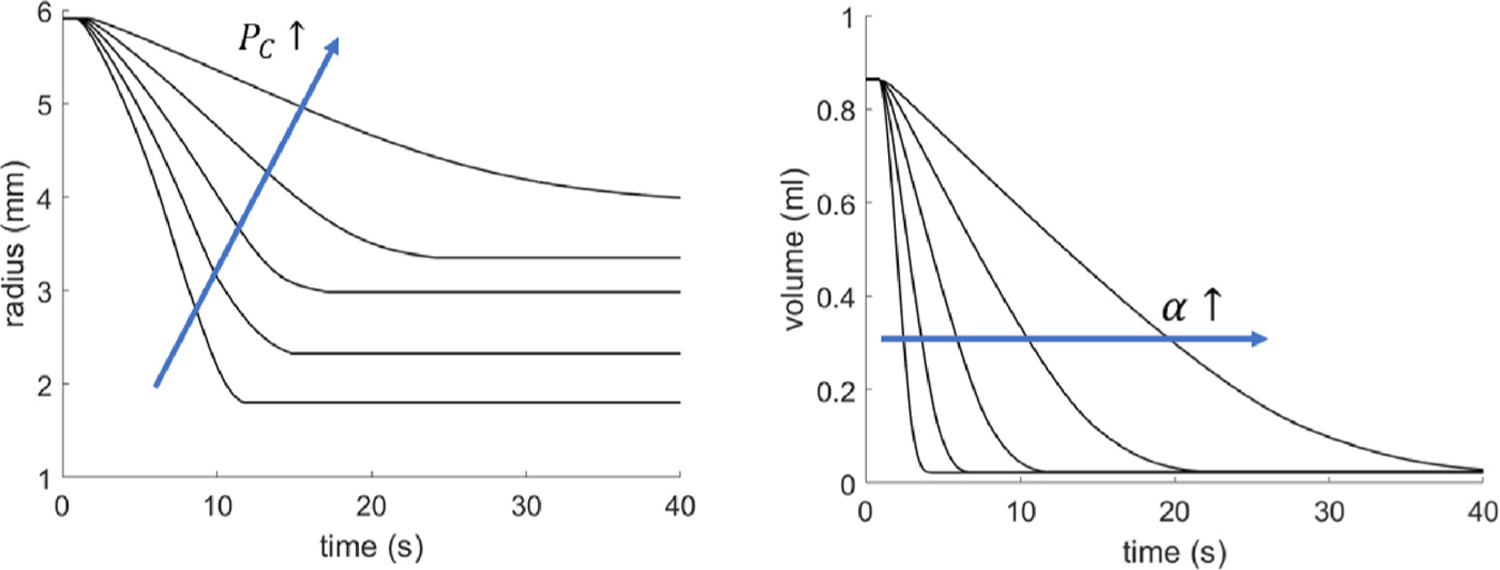
(left) Increasing the cutoff pressure *P*_*c*_ reduces the contractile range (and volume voided) of the bladder; (right) increasing the urethral resistance parameter increases the voiding time.

The micturition model is calibrated to match the mean volume voided and mean void time of the sham bladders, Table 2. This is achieved by tuning two model parameters: the cutoff pressure *P*_*c*_ and urethral resistance parameter *α* (Fig. 10). Given that flow is initiated when the bladder pressure exceeds the cut-off pressure *P*_*c*_, increasing *P*_*c*_ reduces the magnitude of the voiding contraction. Increasing the urethral resistance parameter increases the voiding duration.

### 4.2. Modeling adaptation in response to BOO

To model the G&R response to BOO, two timescales are utilized: a longer time-scale *τ* (days/weeks) for G&R and a shorter-time scale *t* (seconds) for simulating micturition. The bladder model is subject to a constant inflow rate. To facilitate visual illustration of results, we set the inflow to be 0.84ml/day so that the healthy bladder fills and voids once per day. At each time step, as the bladder fills, the updated volume (and radius) of the bladder is computed. The bladder continues to fill until either: voiding is triggered or, if it is unable to void, it experiences the leakage mode. Otherwise, the functional mode is engaged: voiding is triggered, the urodynamic metrics are computed, the bladder is emptied and the cycle of filling begins again. Throughout the simulation, remodeling of recruitment stretches acts to restore collagen/SMC stretches towards their homeostatic values about the onset of voiding. Voiding metrics computed from the micturition model are used to drive SMC growth to restore the volume voided by the bladder (see Eq. (20)). G&R continues until a new homeostasis is achieved and voiding functionality of bladder is restored.

**Fig. 11.**
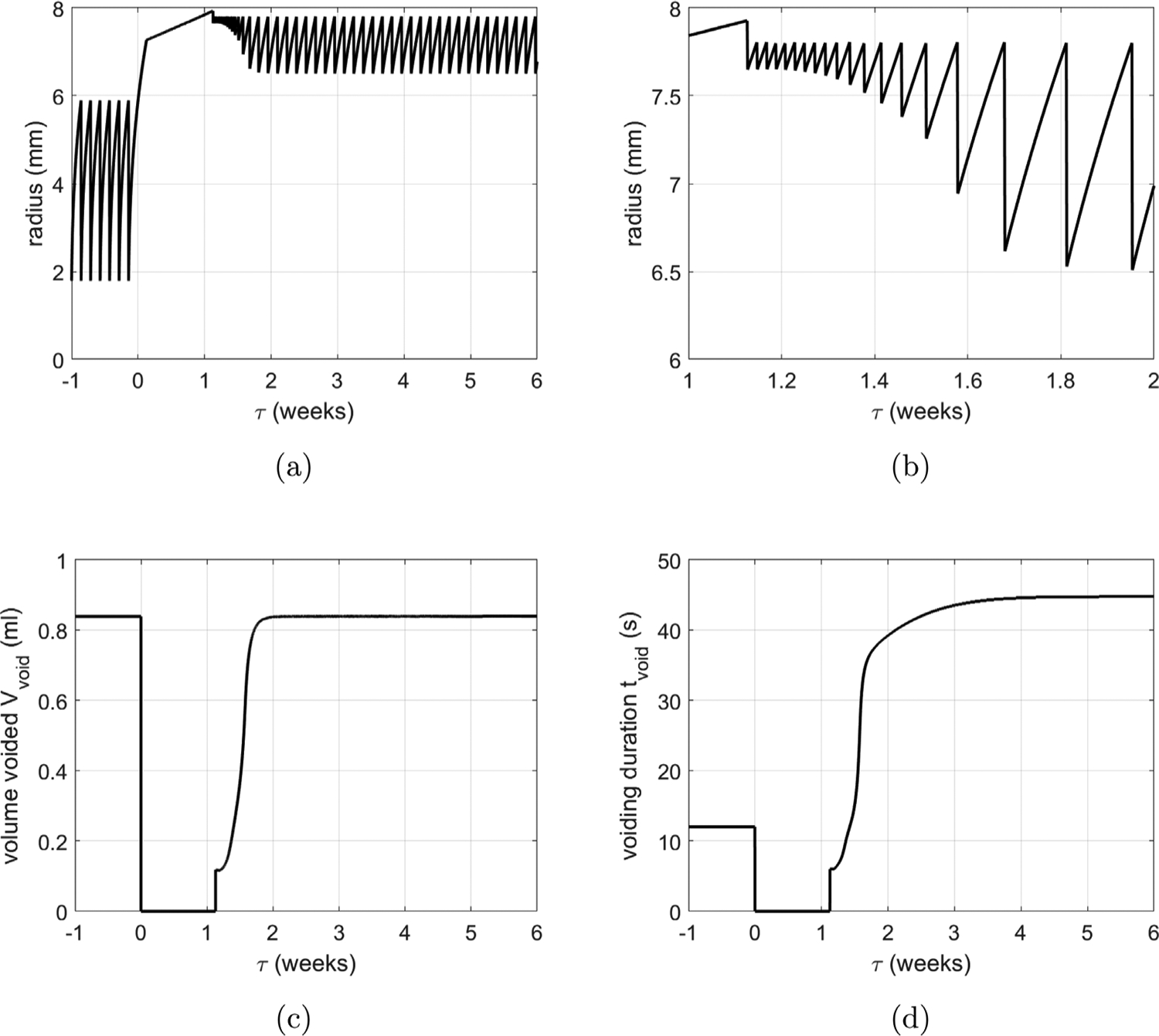
Bladder adaption from pre-obstruction (*t* < 0) to post-obstruction (*t* > 0). (a) Evolution of radius with illustrative filling and voiding cycles and (b) close-up as voiding functionality is restored. Temporal evolution of (c) volume voided and (d) voiding duration.

Fig.11 overviews the voiding behavior of the *in silico* bladder, prior to (*τ* < 0) and following (*τ* ≥ 0) outlet obstruction. The radius of the healthy bladder varies from 2 mm to 6 mm during voiding cycles (Fig. 11a). As the bladder fills and enlarges, voiding is triggered when the muscle stretch reaches 1.5; voiding 0.84 ml takes 12 s, (Fig. 11c,d), and leaves a residual volume of 0.02 ml.

**Fig. 12.**
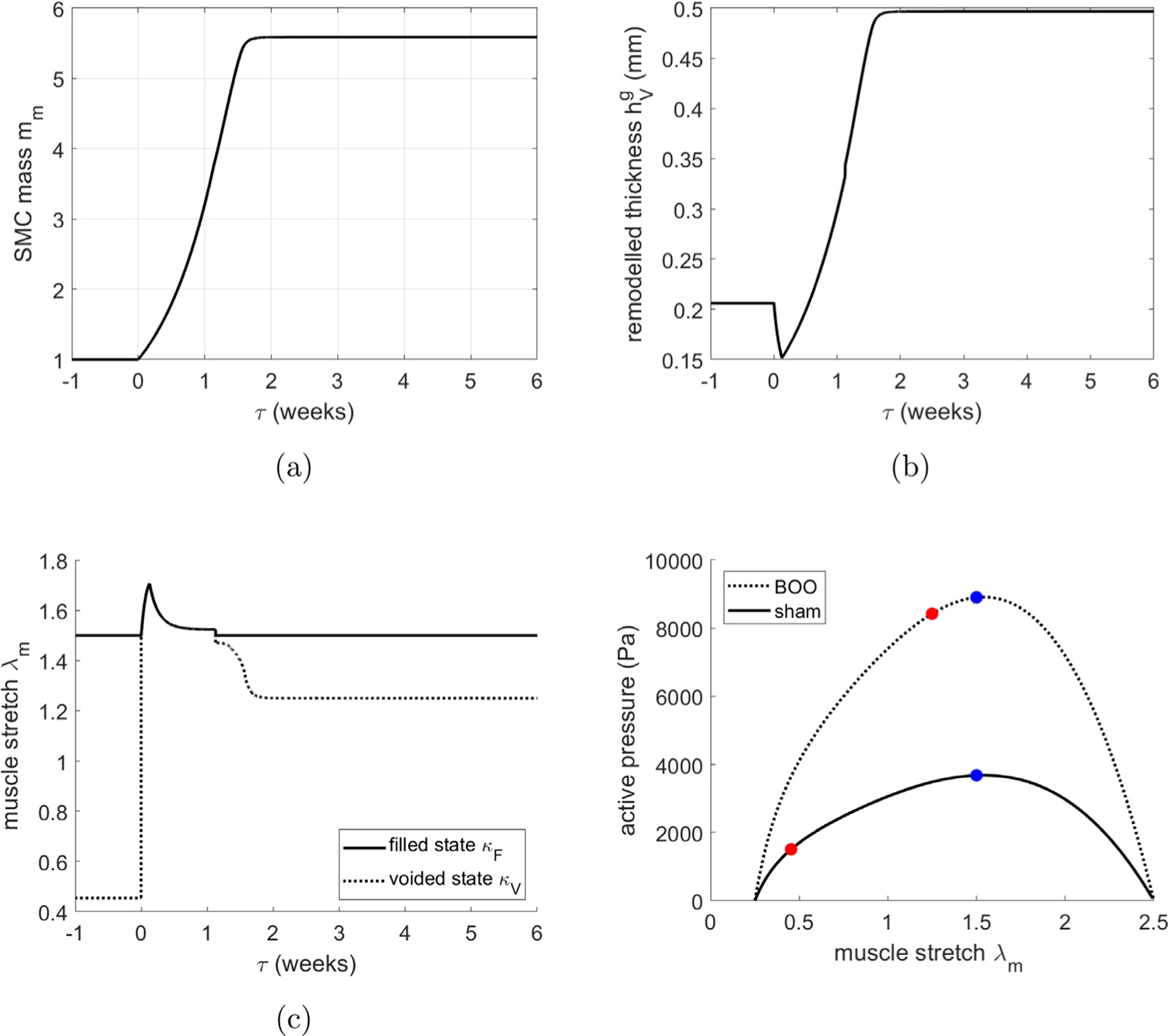
Evolution of (a) SMC mass and (b) tissue thickness at onset of voiding. Evolution of (c) SMC stretch for filled/empty bladder and (d) the relation between muscle stretch and active pressure for sham and BOO bladder models. The blue dots depict the onset of voiding and red dots depict the end of voiding.

For *τ* > 0, we simulate the response to the surgically created outlet obstruction: the cutoff pressure is increased from $P_{c}^{\text{sham}}$ to $P_{c}^{BOO}$ and the urethral resistance parameter is increased from *α*_*sham*_ to *α*_*BOO*_. The G&R response of the CMMG model to obstruction can be broken down into several stages. Immediately following obstruction, the detrusor layer is unable to generate sufficient force to contract the bladder and overcome the increased outlet resistance. The bladder continues to fill and enlarge until its pressure (due to passive elasticity) increases sufficiently to overcome the outlet resistance and generate a leakage flow. Recall, we refer to this stage as the *leaky bladder*; a new steady-state is achieved when the outflow rate equals the filling rate. Subsequently, the *leaky bladder* continues to increase in size (to around 8 mm) as collagen remodels. During this phase, SMCs are remodeling to maintain their stretch towards the target homeostatic stretch (Fig. 12c for 0 < *τ* < 1).

The obstruction drives growth of SMC (Fig. 12a) and is accompanied by thickening of the tissue (Fig. 12b) as the bladder adapts to restore voiding functionality by increasing the active pressure (Fig. 12d). For numerical implementation, we have defined the end of the *leaky bladder* phase as the point when the SMC can generate a contraction in which the voided SMC stretch is less (by 0.02) than the homeostatic SMC voiding stretch. Subsequently, voiding functionality is restored, i.e. the bladder fills and is then triggered to void at *λ*_*m*_ = *λ*_*m*,*h*_. However, at first, the bladder can only void a small volume and hence voiding is more frequent (Fig. 11b) due to the constant filling rate.

At approximately 2 weeks, SMC growth stabilizes and the volume voided is restored. Given that the bladder has enlarged in size, the contraction required to achieve a given void-volume reduces. Hence the variation in radius (Fig. 11a) and range of SMC stretch during voiding decrease (see Figs. 12c and d). Whilst SMC growth stabilizes after 2 weeks, voiding duration does not stabilize until approximately 4 weeks, (Fig. 11d). This is a consequence of collagen continuing to contribute passively to the bladder pressure during voiding. Once collagen remodels to being a protective-sheath about the onset of voiding (by approximately 4 weeks), the voiding time stabilizes to 45 s.

**Fig. 13.**
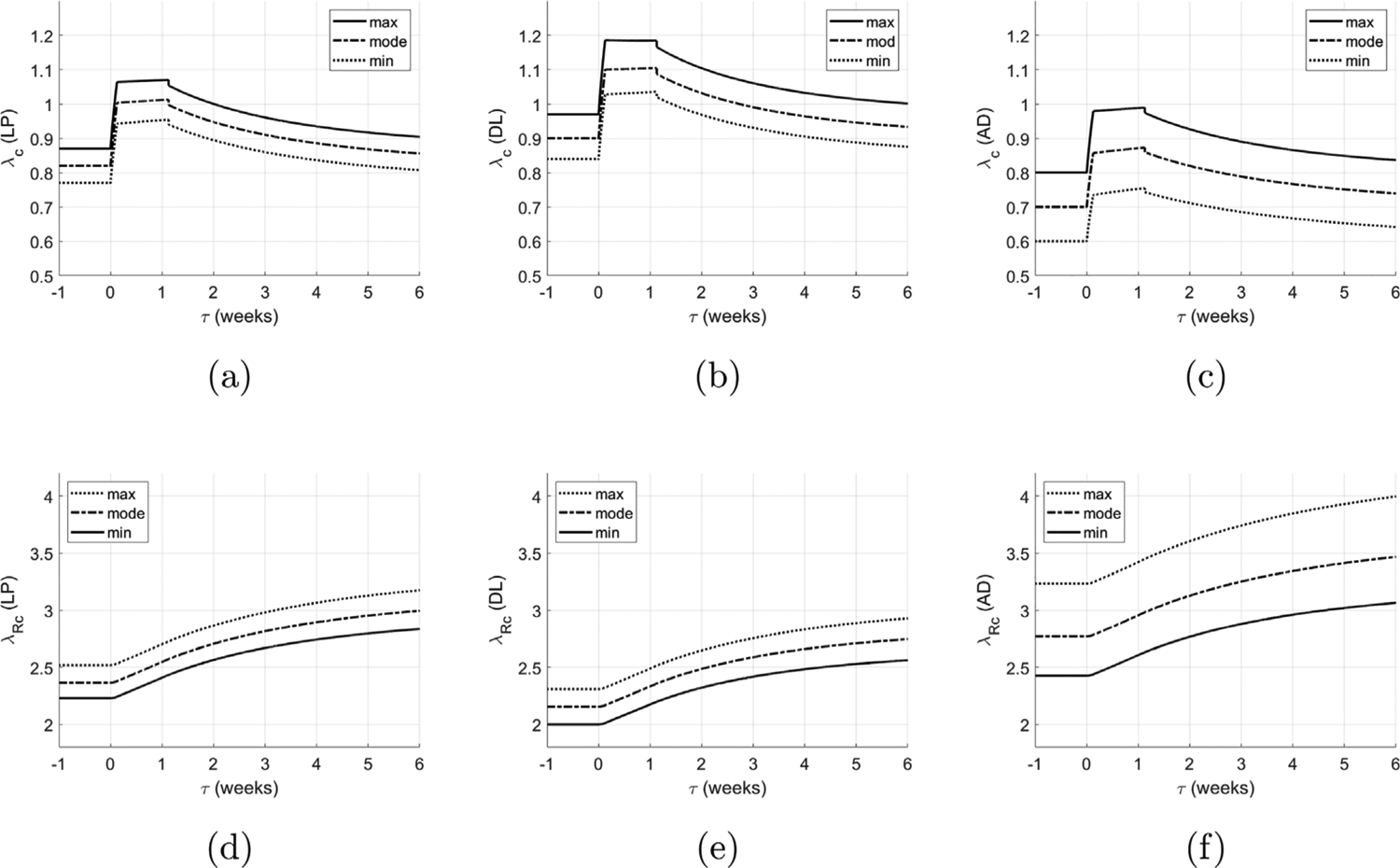
Evolution of collagen fiber stretch distribution (maximum, mode and minimum) for individual layers: (a) lamina propria (b) detrusor and (c) adventitia. Evolution of corresponding collagen recruitment stretch distributions (d)–(f).

**Fig. 14.**
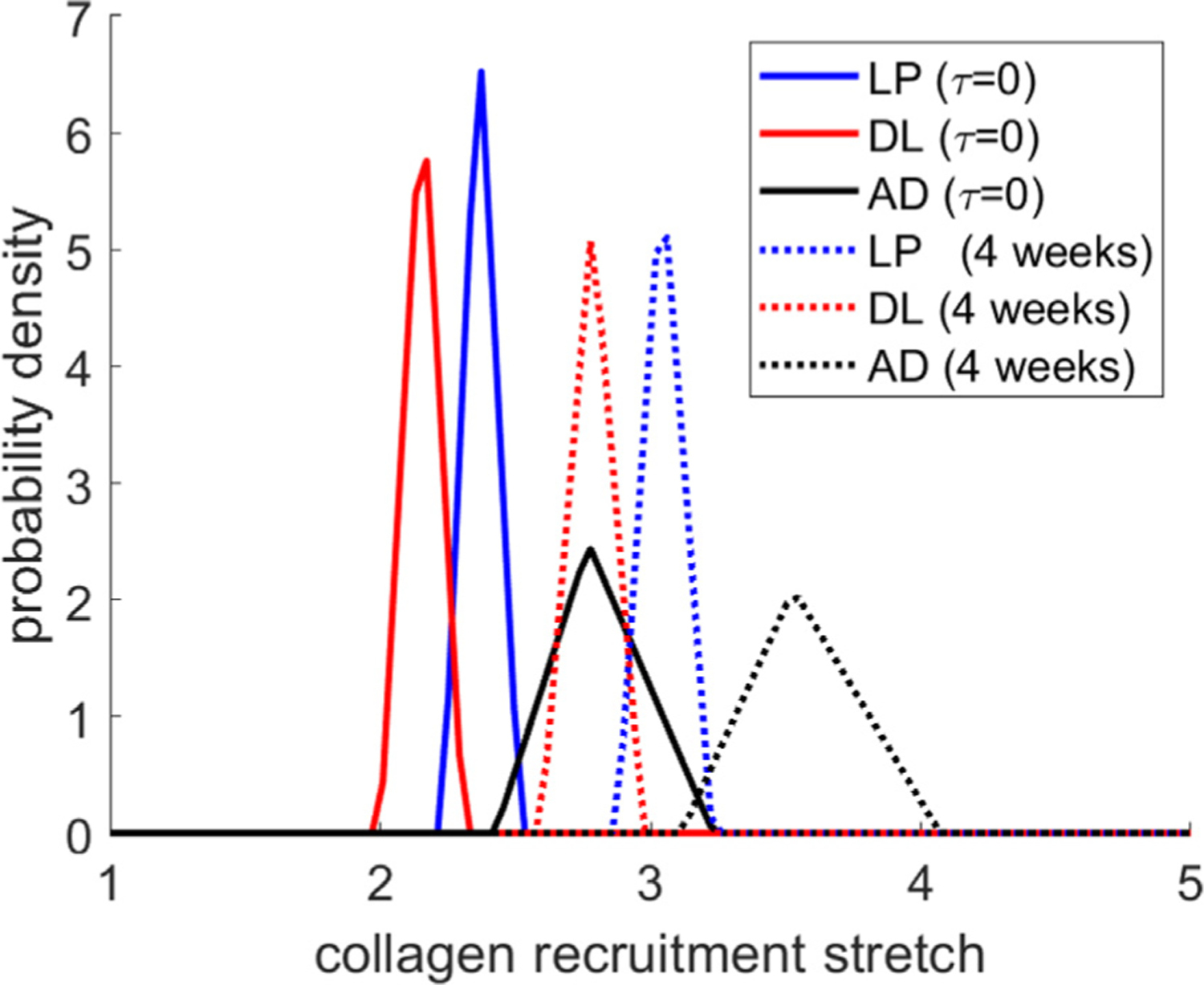
Recruitment stretch distributions in the layers of the bladder for the sham bladder (*t* = 0) and following G&R in response to BOO.

Fig. 13a–c shows the evolution of the collagen fiber stretch distributions in each layer of the bladder relative to the onset of voiding. Initially, the fiber stretch distributions in each layer are equal to the homeostatic distributions and collagen is non-load bearing. Following obstruction, the bladder enlarges and the collagen fibers are engaged in load bearing. Subsequent bladder enlargement is accompanied by collagen remodeling and the stretch distributions remain approximately constant. Once the bladder begins to actively void again, there is a small reduction in radius and an accompanying small drop in fiber stretches. The collagen continues to remodel to restore the collagen stretch distribution towards the deposition stretch distribution (see Fig. 13d–f). This is achieved by a rightward shift of the fiber recruitment distributions as illustrated in Fig. 14.

#### 4.2.1. Predicted urodynamics pre and post obstruction

**Fig. 15.**
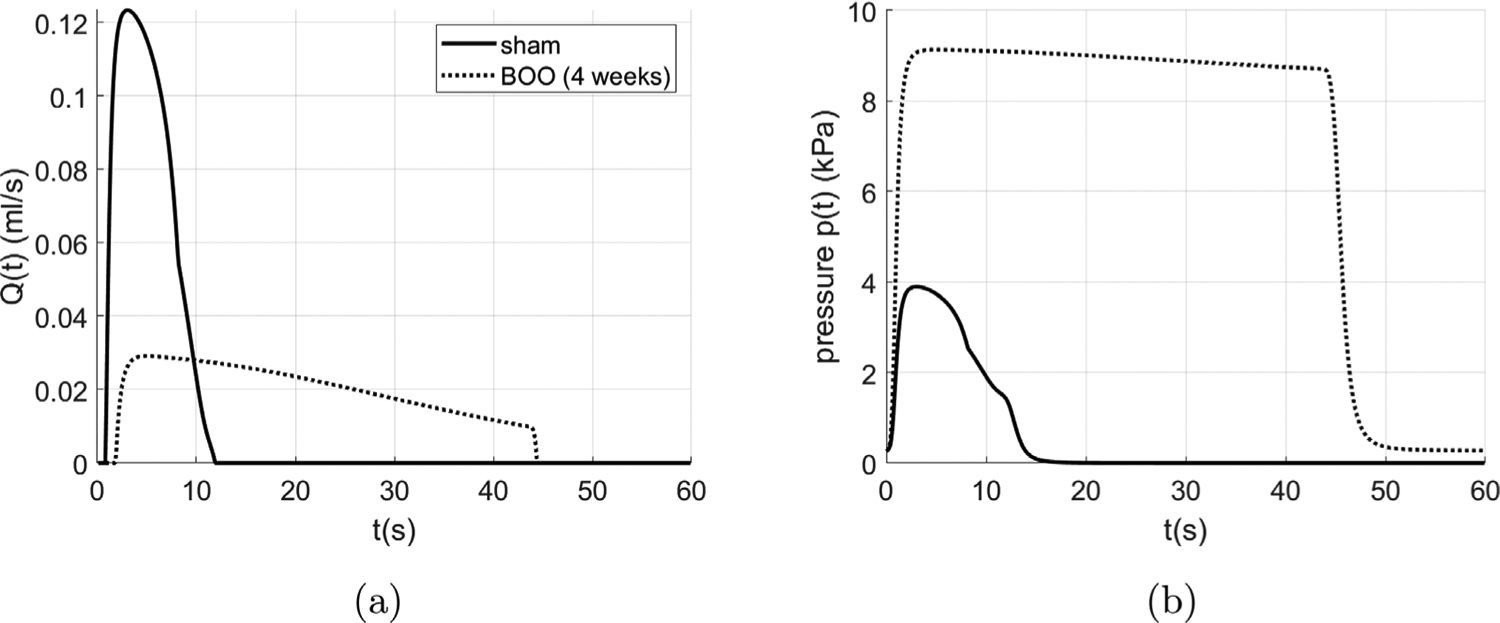
The urodynamic curves of selected time points including (a) time vs. flow rate curve and (b) time vs. pressure curve.

Following obstruction, the maximum flow rate of the bladder decreases by approximately 75% (Fig. 15a) whilst the voiding duration increases from 12 s to 45 s. The maximum voiding pressure more than doubles during voiding for the BOO bladder (Fig. 15b).

### 4.3. Comparison of different growth hypotheses

**Fig. 16.**
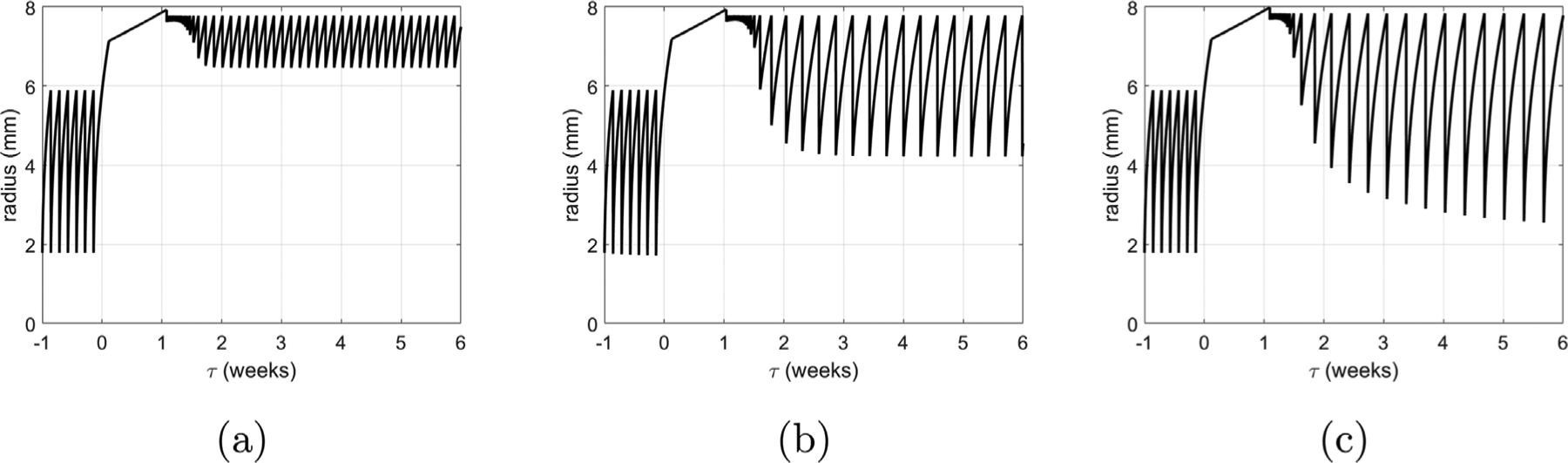
Evolution of radius vs time for different hypothesis of SMC growth (a) SMC growth driven by voided volume driven (b) SMC growth driven by average flow rate (c) SMC growth driven by SMC contraction range.

**Fig. 17.**
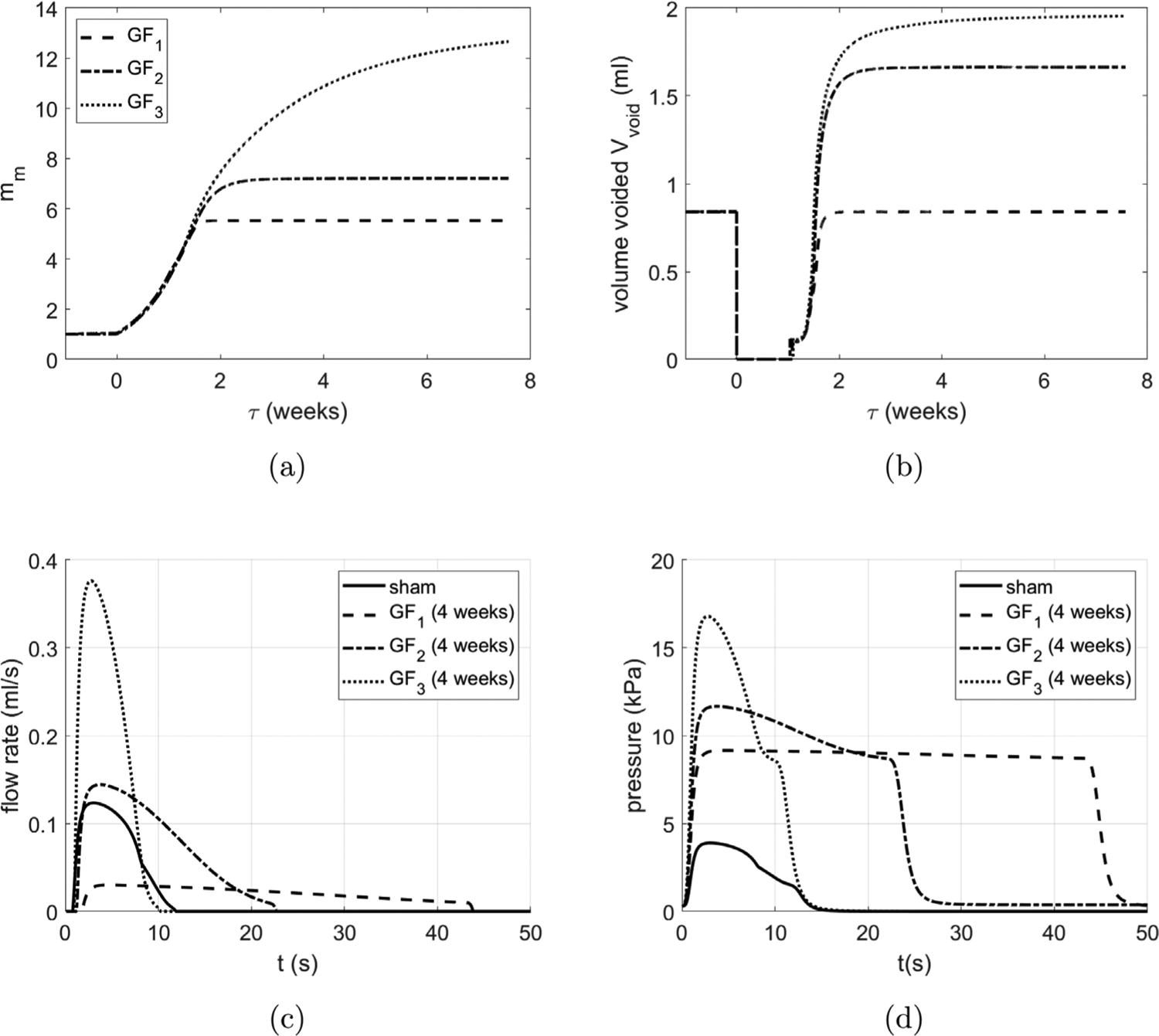
The time vs. (a) SMC mass and (b) volume voided using different SMC growth evolution functions: GF1 - SMC hypertrophy acts to restore volume voided; GF2 - SMC hypertrophy acts to restore average flow rate; GF3 - SMC hypertrophy acts to restore contractile range. Urodynamics (c) and pressure (d) profiles for the three growth cases.

Three illustrate mechanisms to drive SMC hypertrophy are investigated, restoration of: (i) volume voided (as previously used in Section 4.2); (ii) average voiding flow-rate; (iii) contractile range. All cases achieve the same target radius, (Fig. 16). If the bladder adapts to restore average flow rate or contractile range then the volume voided increases, (Fig. 17b). However, significant increases in SMC mass are required to maintain the contractile range (Fig. 17a) and it takes a longer time to achieve homeostasis. Interestingly, if the bladder adapts to maintain contractile range, the voiding time remains approximately the same suggesting a non-pathological adaptation (Fig. 17c), however higher active pressures are needed (17 kPa) (Fig. 17d).

## 5. Discussion

We have presented the first constrained mixture-micturition-growth (CMMG) model of the urinary bladder and used it to simulate the adaptive response of the healthy bladder to BOO. An integrative *in-vivo in vitro in silico* modeling approach underpins the work and facilitates calibration of the healthy bladder model and provides guidance on G&R assumptions. The model provides a mechanistic understanding of how the bladder wall adapts in response to BOO to restore voiding functionality.

### 5.1. Model verification: Comparison between experimental data and model predictions

**Fig. 18.**
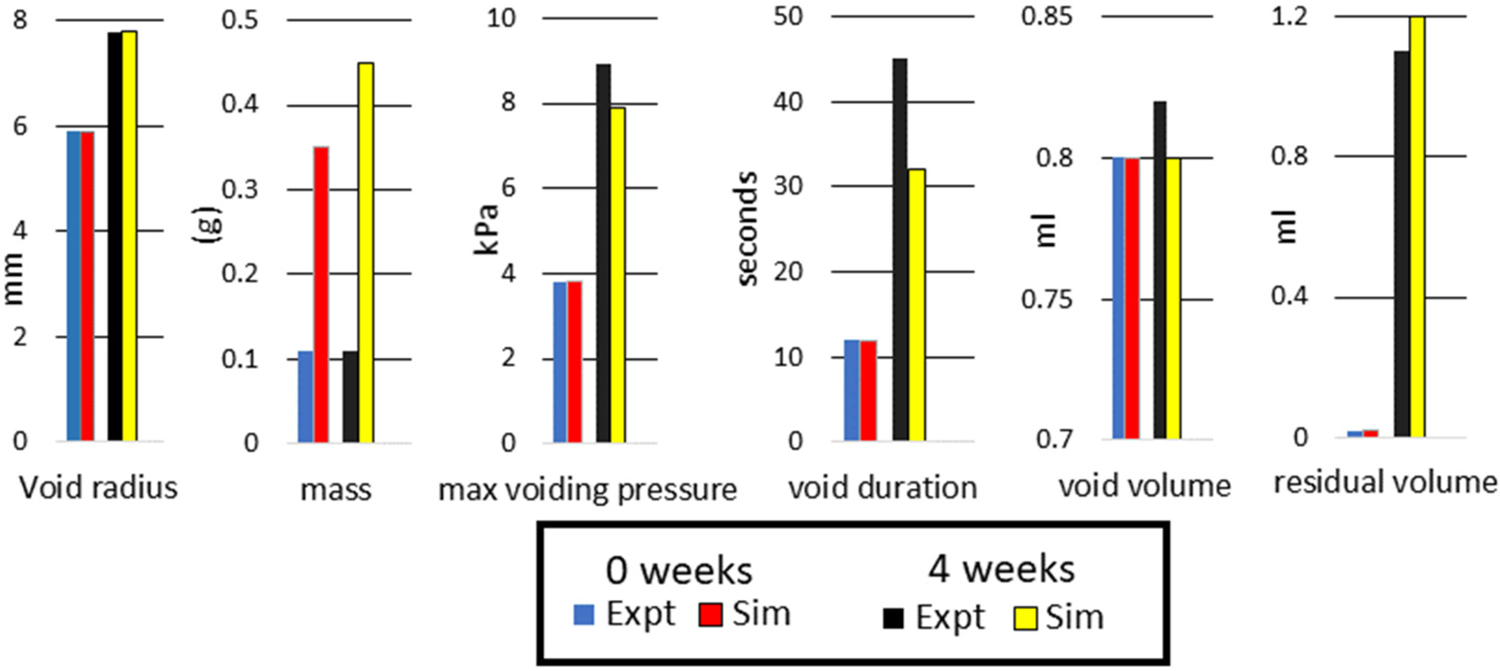
Comparison between *in silico* and experimental model bladder parameters at 4 weeks post-BOO.

Consistent with experimental observations in a rat model of BOO, the model predicts that following initiation of BOO, the bladder enters a leaky state after which hypertrophy of SMC restores the ability to void (Vale et al., 2022). Fig. 18 compares relevant bladder metrics pre-BOO and 4 weeks post-BOO. It can be seen that there is quantitative consistency between model predictions and experiment observations, i.e. the *in silico* bladder: enlarges in size to 7.8 mm; voiding volume is conserved whilst the residual volume increases from 0.02 ml to 1.18 ml; voiding duration increases from 12s to 45 s; maximum voiding pressure increases from 3.9 kPa to around 9 kPa. However, it is evident that the *in silico model* overestimates the increase in bladder mass; 0.45g compared to 0.35g. This latter difference is further discussed below.

**Fig. 19.**
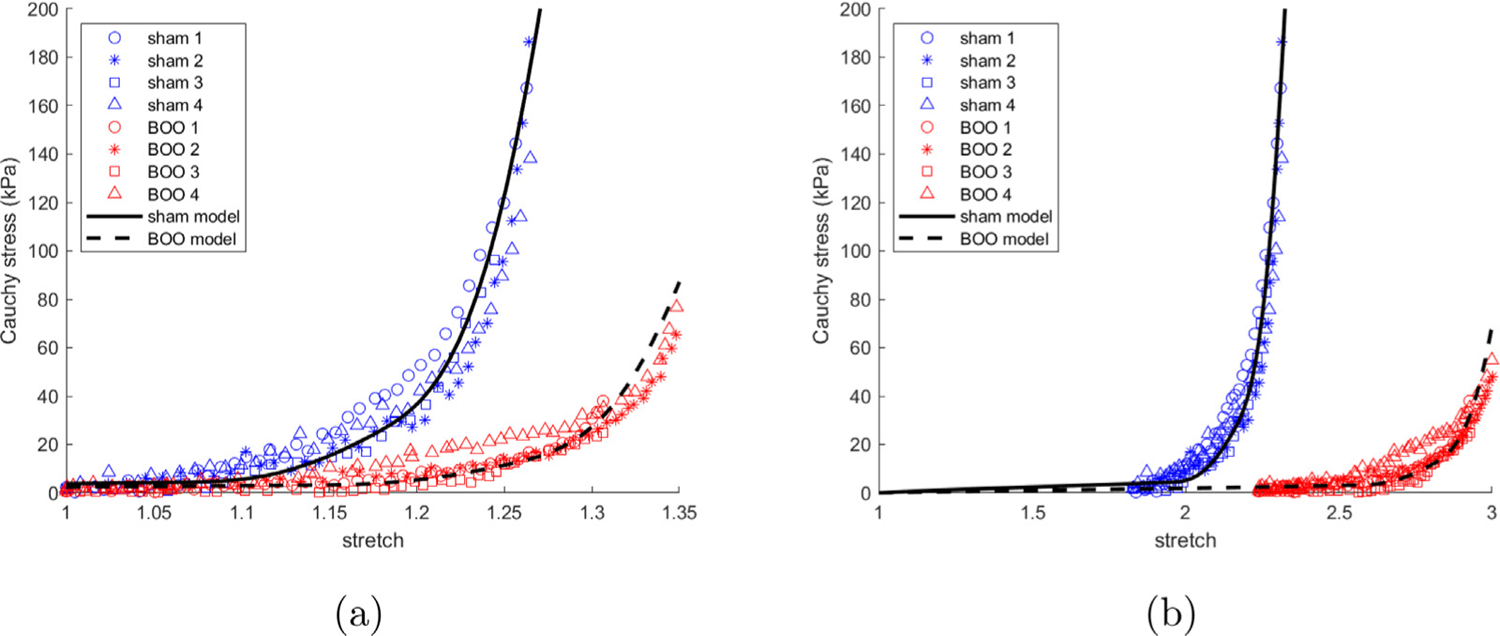
The comparison between experimentally measured mechanical loading curves of sham and 4 week BOO compared with simulation predictions. (a) illustrates the Cauchy stress–stretch relative to the configuration of the biaxial test data whilst (b) shows relative to the unloaded reference configuration of the *in silico* model.

Fig. 19 illustrates the Cauchy stress–stretch experimental data for 4 sham and BOO samples and the relationship for the *in silico model*. For illustration, we have (a) mapped the *in silico* mechanical response relative to the biaxial tissue configuration and conversely (b) mapped the experimental measurements relative to the *in silico* model unloaded reference configuration (this is achieved by aligning data to have consistent voiding stretches, Section 2.6.1). The model is calibrated to the sham; excellent agreement is observed for the sham and for the increase in tissue compliance observed for BOO tissue.

### 5.2. Insights into homeostatic set points for bladder remodeling

While numerous studies have investigated the relationship between mechanical stimuli and SMC remodeling in arteries (Humphrey, 2021) as well as the associated set points for homeostasis, very little is known about this type of coupling in the bladder wall. Here, we used the CMMG model to investigate hypotheses linking SMC growth to bladder biometrics (volume voided, average voiding flow rate, contractile range). The first hypothesis we investigated is that the growth of SMC is driven by volume voided. This is based on our experimental observations that volume voided was restored following hypertrophy. Others have also observed the bladder to increase in size with higher residual volume whilst maintaining volume voided (Nitti et al., 1999). However, whilst it is known that mechanical stress can activate signals that mediate bladder wall hypertrophy, the mechanobiological mechanisms that would enable the bladder to sense how much volume it has voided (to drive SMC growth) remain an open question. We also evaluated the hypotheses that SMC growth evolves to restore either average voiding flow rate or SMC contractile range. However, both these hypotheses led to larger increases in volume voided than observed in experiment. We therefore enlisted the first hypothesis for much of the present work. Nevertheless, we conjecture that flow or SMC stretch sensors may be relevant for adaption in non-pathological conditions, e.g. bladder enlargement during development.

### 5.3. Physiological insights on coupling between remodeling and bladder function

The CMMG model was used to study bladder dysfunction and the remodeling response following BOO surgery. Consistent with experiments (Vale et al., 2022), the *in silico* bladder was unable to void following the increase in outlet resistance, resulting in overflow incontinence after which voiding was recovered. Simulations enable a mechanistic understanding of how this recovery was achieved. Imposition of BOO stimulates rapid SMC hypertrophy. In time, SMC growth is sufficient to generate adequate intravesical pressures to overcome the increased outlet resistance. However, initially, the remodeled bladder voids smaller volumes at higher frequency, compared with the normal bladder. Interestingly, increased void frequency is a clinical symptom of obstructed bladders in humans (Chai et al., 1999). Subsequently, the bladder transitions to the compensated phase as SMC growth stabilizes in response to recovery of voiding function.

### 5.4. Clinical need-diagnosis

The clinical diagnosis of BOO in the context of a non-neurogenic history, involves taking a detailed urological history and assessing the lower urinary tract symptoms including storage, voiding and post-voiding symptoms. A variety of symptom score questionnaires are available (Anon, 2022) including the International Prostate Symptom Score, the International Consultation on Incontinence questionnaire, and the Danish Prostate Symptom Score. There is strong evidence that a validated symptom score questionnaire should be used. More generally, the mainstay of assessment of lower urinary tract symptoms (LUTS) is the use of a bladder diary to assess frequency of voiding and the volumes of urine produced, ideally recorded over a consecutive three-day period. Following a clinical examination and analysis of the urine, the use of a blood test to exclude cancer (prostate specific antigen), a post-voiding residual to check that the bladder is emptying to completion and where appropriate a flow test to assess the flow of urine from the bladder through the urethra. In this work, we used our CMMG model to understand how changes to the bladder wall lead to changes clinical parameters such as maximum active bladder pressure, contractile range, void time, residual volume and voided volume during the G&R response to BOO. In the future, simulations of this kind could be extended to other types of data available in clinical evaluations in order to more directly impact clinical practice.

### 5.5. Relevance of the BOO animal model

The animal model of BOO induced by partial urethral ligation has most commonly been used to investigate the pathophysiology of male LUTS associated with BOO resulted from BPH. However, the majority of previous basic research studies on BOO have utilized female animals. Motivated by the application to BPH, this project used male rats to produce the BOO condition underlying male LUTS (Zvara et al., 2002). A recent study also reported that this male BOO model exhibits the early hypertrophy-compensation phase followed by the later decompensation phase of bladder dysfunction, similar to those observed in human BPH/BOO (Shinkai et al., 2020).

### 5.6. Need for a combined in vitro –in vivo–in silico approach

Animal models may also provide the critical data needed to overcome limitations in current diagnosis and treatment practices. For example, urodynamics is the conventional approach for diagnosing and quantifying the severity of BOO prior to surgery. However, whilst it has been considered the “gold standard” evaluation for BOO and is used as a predictor of outcome to surgical intervention to relieve the outlet obstruction, recent work in a randomized study looking at the role of urodynamics prior to surgery (Bailey et al., 2015) raised concerns over its usefulness. We conjecture that a more mechanistic understanding of the relationship between the urodynamic data and progressive changes to the bladder wall during the three stages of BOO pathology is essential for improving the clinical utility of urodynamics data. Such an understanding requires a modeling approach of the kind developed here that integrates a model of filling/voiding with a longer time scale G&R model. Moreover, we envisage future work that leverages the *in vivo in vitro in silico* approach introduced here will enable the design of new diagnostic tools for assessing bladder dysfunction and provide guidance on developing new treatments.

### 5.7. Model critique and future directions

Experimental data confirms the bladder undergoes three stages of remodeling in response to BOO: an initial *hypertrophy* phase, followed by a *compensation* phase, and finally, a *decompensation* phase (Fusco et al., 2018). The hypertrophy stage consists of SMC growth accompanied by angiogenesis to meet the increased metabolic demands of the tissue. During the compensation stage, the bladder maintains effective voiding function but is subject to cyclic ischaemia-reperfusion injury; this leads to matrix accumulation (fibrosis) and also neuronal loss (Levin et al., 2004) that is accompanied by diminished SMC contractility. Finally, in the decompensation stage, SMC atrophy occurs leading to loss of bladder functionality. In the present work, we only model the hypertrophy and early compensation stages of BOO- without ischaemic damage. However, the CMMG model provides foundations for developing a more complete representation of all stages of BOO. We envisage such a model may assist in designing/evaluating pharmacological or surgical interventional strategies for clinical management of the disease.

In the present work, the CMMG model was applied to an idealized spherical membrane model with an isotropic response from wall components, so that the coupling between wall remodeling and the voiding/filling process could be studied. In future work, this approach can be applied to more realistic bladder models that account for the bladder’s spatially heterogeneous material properties (Morales-Orcajo et al., 2018; Trostorf et al., 2021) and complex anatomical geometry. Furthermore, we have not accounted for external body forces on the bladder, e.g. from abdominal organs or diaphragmatic pressure. These can also influence loading on the bladder.

We used a simple time-dependent activation function *S*(*t*) to control the active stress of SMCs during voiding. This allows us to simulate the pressure/flow waveforms which provide metrics that feed into G&R algorithms to adapt the bladder to maintain voiding functionality. The model could be sophisticated to incorporate electro-chemical activity, e.g. as in Seydewitz et al. (2017), however, at this stage our interest is to understand the regulatory processes that govern homeostasis and G&R of the constrained mixture.

The growth model accounted for the increase in mass and active stress generated by SMCs. Whilst we obtained consistency with the increased compliance of the tissue and urodynamics metrics, the model overestimated the increase in bladder mass, Fig. 18. We have assumed that SMC tension increases in proportion to the increase in SMC. This may not be true, leading to inaccuracies in the predicted increase in SMC mass required to yield a given increase in active tension. In future studies, the changes in relative mass fraction of the components and wall layers could be measured, providing guidance for advancing the model.

Future developments of the model will build on existing computational growth models that account for anatomical geometries and anisotropic wall properties (Teixeira et al., 2020), dynamic elasticity of the SMCs (Speich et al., 2006, 2009; Duval et al., 2021), elastogenesis (Heise et al., 2012), volumetric growth (Grytsan et al., 2017); and in-corporation of regulatory fibrotic pathways for collagen growth (Aparício et al., 2016). Consideration of the molecular aspects of the remodeling, (for example, those associated with ischaemia) during the compensation, decompensation stages of BOO will provide insights on the reversibility of pathological changes associated with BOO.

## 6. Conclusion

We presented a novel CMMG model for the bladder’s adaptive G&R response to outlet obstruction. The model was calibrated to experimental data and predictions were consistent with *in vivo* experiments of bladder outlet obstruction. This work is an important step towards the development of patient specific *in silico* models of the bladder that can predict changes to bladder functionality and hence guide the selection and timing of patient treatment. We envisage these models can be leveraged in the future so clinicians can make more effective use of diagnostic data and researchers can design new pharmacological and surgical interventions.

## Figures and Tables

**Table 1:** Summary of definitions of cystometric parameters.

	Bladder pressures
*P*_*basal*_	Basal pressure; Passive pressure in the empty bladder, taken to be lowest pressure in cystometry
$P_{\text{passive}}^{\text{max}}$	Pressure immediately after reflex contraction
$P_{\text{void}}^{\text{max}}$	Peak pressure
$P_{\text{act}}^{\text{max}}$	Maximum active voiding pressure $P_{\text{void}}^{\text{max}}-P_{\text{passive}}^{\text{max}}$
*P*_*c*_	Cut-off pressure to initiate flow
	Bladder volumes
*V*_*o*_	Volume of harvested (unloaded) bladder, (estimated from dimensions)
*V*_*res*_	Post-void residual volume, measured in cystometry
*V*_*void*_	Voided volume, measured in cystometry
*V*_*F*_	Filled volume (*V*_*F*_ = *V*_*res*_ + *V*_*void*_)

**Table 2:** In vivo measured urodynamic parameters and mass for sham (n=12) and BOO (n=12) bladders 4 weeks post surgery.

Quantities	Symbol	Sham	BOO
Void volume (*ml*)	*V*_*void*_	0.84 ± 0.03	0.82 ± 0.12
Residual volume (*ml*)	*V*_*res*_	0.02 ± 0.008	1.2 ± 0.23
Void duration (s)	*t*_*void*_	12 ± 0.4	45 ± 5.0
Max filling pressure (Pa)	$P_{\text{passive}}^{\text{max}}$	300 ± 80	280 ± 70
Max void pressure (Pa)	$P_{\text{void}}^{\text{max}}$	3900 ± 220	8900 ± 640
Mass (g)	*M*_*B*_	0.11 ± .005	0.35 ± 0.041

**Table 3:** Dimensions of the explanted (unloaded) Sham (n=4) and BOO bladders (n=4)

Sham	S1	S2	S3	S4	Average
Diameter *D*_0_ (mm)	4.31	5.05	4.76	4.84	4.74 ± 0.16
Height *Y*_0_ (mm)	11.10	10.32	9.64	9.36	10.10 ± 0.39
Wall thickness *H*_0_ (mm)	0.72	0.71	0.85	0.82	0.78 ± 0.04
BOO	B1	B2	B3	B4	Average
Diameter *D*_0_ (mm)	7.92	8.40	9.38	10.08	8.95 ± 0.49
Height *Y*_0_ (mm)	19.14	17.39	13.75	15.35	16.41 ± 1.18
Wall thickness *H*_0_ (mm)	1.61	1.53	1.98	1.10	1.56 ± 0.18

**Table 4:** Measurements of onset of recruitment of collagen fibers in the lamina propria (LP) and detrusor layers (DL) of Sham (n=4) and BOO (n=4) bladder samples, obtained from MPM images. The parameter ${\bar{\lambda }}_{F}$ is the computed voiding stretch for each sample.

Sham	S1	S2	S3	S4	Average
${\bar{\lambda }}_{R}^{\text{min}}\;(\text{LP})$	1.06	1.21	1.21	1.19	1.17 ± 0.04
${\bar{\lambda }}_{R}^{\text{min}}\;(\text{DL})$	1.07	1.12	1.05	1.06	1.08 ± 0.02
${\bar{\lambda }}_{F}$	1.06	1.04	1.07	1.02	1.05 ± 0.01
BOO	B1	B2	B3	B4	Average
${\bar{\lambda }}_{R}^{\text{min}}\;(\text{LP})$	1.26	1.30	1.25	1.16	1.27 ± 0.02
${\bar{\lambda }}_{R}^{\text{min}}\;(\text{DL})$	1.14	1.14	1.15	1.08	1.13 ± 0.02
${\bar{\lambda }}_{F}$	1.07	1.03	1.17	1.06	1.05 ± 0.01

**Table 5:** Mean fiber deposition stretch distributions in the LP and DL of sham/BOO bladders.

Bladder	$\text{LP: }\lambda_{c,h}^{\text{min}},\lambda_{c,h}^{\text{mode}},\lambda_{c,h}^{\text{max}}$	$\text{DL: }\lambda_{c,h}^{\text{min}},\lambda_{c,h}^{\text{mode}},\lambda_{c,h}^{\text{max}}$
Sham (mean)	0.77, 0.82, 0.87	0.84, 0.90, 0.97
BOO (mean)	0.77, 0.80, 0.86	0.84, 0.90, 0.96

**Table 6:** CMMG parameters used in the simulations in Section 4. The rational for these choices is given in Appendix B.

Parameter	Meaning	Value
*R*_*V*_, *R*_0_, *R*_*F*_	Voided/unloaded/filled radii	1.7/3.0/5.9 mm
$\lambda_{F}^{\left(0\right)}$	Initial stretch *(R*_*F*_ / *R*_0_*)*	1.94
*k*_*nc*_	Isotropic material parameter	1.17 kPa
*k*_*c*_ (*LP*)	Collagen material parameters	11.7 MPa
*k*_*c*_ (*DL*)	In each layer	0.79 MPa
*K*_*c*_ (*AD*)		11.7 MPa
$\lambda_{c,h}^{q}(LP)$	Collagen homeostatic stretches	0.77, 0.82, 0.87
$\lambda_{c,h}^{q}(DL)$	In each layer	0.84, 0.90, 0.97
$\lambda_{c,h}^{q}(AD)$	(*q*= min/mode/max)	0.6, 0.7, 0.8
$k_{m}^{\text{act}}$	SMC active modulus	5770 Pa
$\lambda_{m}^{\text{min}}$	SMC active stress parameter	0.25
$\lambda_{m}^{\text{max}}$	SMC active stress parameter	2.5
*λ*_*m*,*h*_	SMC homeostatic stretch	1.5
$P_{c}^{\text{sham}}$	Cutoff pressure sham	1450 Pa
$P_{c}^{\text{BOO}}$	Cutoff pressure BOO	8487 Pa
*α*_*sham*_	Urethral resistance sham	19800 Pa/(ml/s)
*α*_*BOO*_	Urethral resistance BOO	22075 Pa/(ml/s)
*Q*_*in*_	Bladder filling rate	0.8 ml/day
*α*_*c*_	Collagen remodeling rate	3.6
*α*_*m*_	Muscle remodeling rate	40
*β*_*m*_	Muscle growth rate	5

## Appendix A. Material parameters from the biaxial experiments

Table A.7 shows best-fit parameters for fitting the constitutive model to the biaxial Cauchy stress–stretch data obtained from experiments on four sham and four BOO tissue specimens. As inputs, the fitting algorithm used the Cauchy stress–stretch data and the (measured) minimum values of collagen recruitment ${\bar{\lambda }}_{R}^{\text{min}}$ measured for the LP and DL layers and the void stretch ${\bar{\lambda }}_{F}^{\text{void}}$. A value for *k*_*nc*_ was determined by assuming only the neo-Hookean components were load bearing at the onset of voiding. The five free material parameters in the fitting procedure were the width and skew of the collagen recruitment distributions in each layer and the ratio of the collagen stiffnesses $\left(k_{c}^{LP}/k_{c}^{DL}\right)$.

Results for the triangular distribution function for individual samples, Table A.7, are shown in Fig. A.20. For the sham bladder, the DL collagen recruited before LP collagen. There was overlap of recruitment stretches giving a bimodal recruitment distribution for collagen. The width of recruitment tends to be greater for DL than for LP. Similarly for the BOO bladder, DL collagen recruited earlier than LP collagen, again resulting in a bimodal distribution of recruitment stretch (see Table A.8).

## Appendix B. CMMG model: Parameterization

The rational behind the choice of model parameters in Table 6 is outlined in this appendix. Briefly, The steps are as follows:

- The unloaded (*R*_0_), filled (*R*_*F*_) and voided (*R*_*V*_) radii were computed from the mean unloaded, filled and residual bladder volumes of the sham bladders given in (see Table 2) using *R* = (3*V*/4*π*)^1/3^.
- The tissue stretches at filled and voided configurations were then calculated from *λ*_*F*_ = *R*_*F*_*/R*_0_ and *λ*_*V*_ = *R*_*V*_*/R*_0_, respectively.
- The collagen is configured to be non load bearing at the onset of voiding. Hence the parameter *k*_*nc*_ can be analytically determined from the force-balance equation given the stress function *σ*_*nc*_ for the non-collagenous constituents where for the deformation considered *σ*_*nc*_ = 4*k*_*nc*_*λ*^2^(1 − 1/*λ*^6^) (Watton et al., 2009a).
- Collagen material parameters: $k_{c}^{DL}$ was taken to be mean value obtained from biaxial data; $k_{c}^{LP}$ was taken to be product of the mean $k_{c}^{DL}$ and the mean $k_{c}^{LP}/k_{c}^{DL}$ ratio.
- The homeostatic collagen deposition stretch distribution parameters $\left(\lambda_{c,h}^{\text{min}},\lambda_{c,h}^{\text{mod}}\lambda_{c,h}^{\text{max}}\right)$ for the LP and DL were informed from the *vitro* studies, Table 5.
- The initial values for the recruitment stretches $\left(\lambda_{Rc}^{\text{min}},\lambda_{Rc}^{\text{mod}}\lambda_{Rc}^{\text{max}}\right)$ are determined from the mean values of collagen deposition stretches and the initial stretch at the filled state, $\lambda_{F}^{\left(0\right)}=R_{F}/R_{0}$, using Eq. (18). The recruitment stretches evolve (see Eq. (17)) to maintain the collagen stretch distribution at the onset of voiding (*κ*_*F*_) towards the homeostatic deposition stretch distribution.
- We choose active SMC parameters $\lambda_{m}^{\text{min}}=0.25$, $\lambda_{m}^{\text{max}}=2.5$ and the homeostatic SMC stretch at the onset of void to be to the left of the maximum of the active-pressure SMC stretch relationship *λ*_*m*,*h*_ = 1.5. Using this function and choice of parameters, the bladder model can simulate the contractile range of the sham bladder (*R*_*F*_*/R*_*V*_), the active pressure decreases monotonically as the stretch decreases and the bladder maintains a consistent pressure over a large voiding range, compare the pressure–volume loop for the model (Fig. 9) with Fig 1B in Peng et al. (2020).
- The initial value of the muscle recruitment stretch was set to $\lambda_{R}^{m}=\lambda_{F}^{\left(0\right)}/\lambda_{m,h}$.
- $k_{m}^{\text{act}}$ is determined from the active-pressure stretch relation, Eq. (25), with $p_{\text{act}}=P_{\text{act}}^{\text{max}}$ (see Table 1) and *λ*_*m*_ = *λ*_*m*,*h*_. The parameters of the stimulus function *S*(*t*) are taken to be *k*_*m*1_ = 1 and *k*_*m*2_ = 1; these values are chosen so that the increase of *S*(*t*) to 1 and later decrease to 0 only represents a small fraction of the voiding cycle. To trigger the decay of *S*(*t*) towards the end of voiding, we choose $Q_{\text{crit}}^{S}$ = to be 1/20 of the maximum flow rate.
- The cutoff pressures, $P_{c}^{\text{sham}}$ and $P_{c}^{\text{BOO}}$, are determined so that the bladder achieves the desired contractile range during voiding. Briefly, at the tissue voided stretch *λ*_*V*_, we require the flow-rate *Q*(*t*) = 0. Hence from Eq. (13), we solve $p_{\text{active}}\left(\lambda_{m}\left(\lambda_{V},\lambda_{Rm}\right)\right)-P_{c}^{\text{sham}}=0$ (and similarly for $P_{c}^{\text{BOO}}$).
- The urethral resistances, *α*_*sham*_, *α*_*BOO*_, are determined by matching the experimental voiding duration.
- The collagen remodeling rate *α*_*c*_ is numerically determined so that the radius matches the experimental value when voiding is restored. The SMC remodeling rate *α*_*m*_ is chosen so that the SMC rapidly remodels to restore its stretch towards the homeostatic stretch within 1 week. The muscle growth rate *β*_*m*_ is numerically determined so that the SMC growth can restore the volume voided within four weeks.

**Table A.7:** Fitted material and microstructural parameters for the sham and BOO tissue samples using data from biaxial testing.

Sham	*k*_*nc*_ (kPa)	$k_{c}^{LP}$ (MPa)	$k_{c}^{DL}$ (MPa)	$\lambda_{R}^{\text{min}},\lambda_{R}^{\text{mode}},\lambda_{R}^{\text{max}}$ (LP)	$\lambda_{R}^{\text{min}},\lambda_{R}^{\text{mode}},\lambda_{R}^{\text{max}}$ (DL)	rsq
S1	17.9	9.79	0.70	1.19, 1.27, 1.39	1.07, 1.12, 1.25	0.998
S2	19.46	13.90	0.70	1.21, 1.26, 1.33	1.12, 1.16, 1.28	0.998
S3	10.62	13.19	1.16	1.21, 1.26, 1.40	1.07, 1.21, 1.25	0.996
S4	24.25	7.39	0.42	1.19, 1.28, 1.33	1.06, 1.17, 1.21	0.998
BOO	*k*_*nc*_ (kPa)	$k_{c}^{LP}$ (MPa)	$k_{c}^{DL}$ (MPa)	${\bar{\lambda }}_{R}^{\text{min}},{\bar{\lambda }}_{R}^{\text{mode}},{\bar{\lambda }}_{R}^{\text{max}}$ (LP)	${\bar{\lambda }}_{R}^{\text{min}},{\bar{\lambda }}_{R}^{\text{mode}},{\bar{\lambda }}_{R}^{\text{max}}$ (DL)	rsq
B1	6.41	2.64	0.13	1.26, 1.30, 1.36	1.14, 1.23, 1.24	0.990
B2	6.86	3.66	0.40	1.30, 1.35, 1.41	1.14, 1.29, 1.36	0.993
B3	1.10	0.31	0.31	1.25, 1.47, 1.50	1.15, 1.17, 1.37	0.982
B4	8.78	1.59	0.08	1.25, 1.35, 1.36	1.08, 1.10, 1.18	0.983

**Table A.8:** Fiber deposition stretch distributions in the LP and DL of sham and BOO bladders. Parameters are determined from (fitted) recruitment stretches (Table A.7) and the voiding stretch ${\bar{\lambda }}_{F}$ (Table 4).

Sham	$\text{LP: }\lambda_{c,h}^{\text{min}},\lambda_{c,h}^{\text{mode}},\lambda_{c,h}^{\text{max}}$	$\text{DL: }\lambda_{c,h}^{\text{min}},\lambda_{c,h}^{\text{mode}},\lambda_{c,h}^{\text{max}}$
S1	0.76, 0.84, 0.89	0.85, 0.95, 0.99
S2	0.78, 0.82, 0.86	0.82, 0.90, 0.93
S3	0.77, 0.85, 0.89	0.86, 0.89, 1.00
S4	0.77, 0.80, 0.85	0.84, 0.87, 0.96
Mean	0.77, 0.82, 0.87	0.84, 0.90, 0.97
BOO	$\text{LP: }\lambda_{c,h}^{\text{min}},\lambda_{c,h}^{\text{mode}},\lambda_{c,h}^{\text{max}}$	$\text{DL: }\lambda_{c,h}^{\text{min}},\lambda_{c,h}^{\text{mode}},\lambda_{c,h}^{\text{max}}$
B1	0.79, 0.82, 0.85	0.86, 0.87, 0.94
B2	0.73, 0.76, 0.80	0.76, 0.80, 0.91
B3	0.78, 0.79, 0.94	0.85, 1.00, 1.02
B4	0.78, 0.79, 0.85	0.90, 0.97, 0.98
Mean	0.77, 0.80, 0.86	0.84, 0.90, 0.96
